# RRE-Finder: a Genome-Mining Tool for Class-Independent RiPP Discovery

**DOI:** 10.1128/mSystems.00267-20

**Published:** 2020-09-01

**Authors:** Alexander M. Kloosterman, Kyle E. Shelton, Gilles P. van Wezel, Marnix H. Medema, Douglas A. Mitchell

**Affiliations:** a Department of Molecular Biotechnology, Institute of Biology, Leiden University, Leiden, The Netherlands; b Department of Chemistry, University of Illinois at Urbana-Champaign, Urbana, Illinois, USA; c Carl R. Woese Institute for Genomic Biology, University of Illinois at Urbana-Champaign, Urbana, Illinois, USA; d Department of Microbial Ecology, Institute of Ecology (NIOO-KNAW), Wageningen University, Wageningen, The Netherlands; e Bioinformatics Group, Wageningen University, Wageningen, The Netherlands; City of Knowledge

**Keywords:** RRE, RiPPs, bioinformatics, genome mining, natural products, secondary metabolism, Web tool

## Abstract

Many ribosomally synthesized and posttranslationally modified peptide classes (RiPPs) are reliant on a domain called the RiPP recognition element (RRE). The RRE binds specifically to a precursor peptide and directs the posttranslational modification enzymes to their substrates. Given its prevalence across various types of RiPP biosynthetic gene clusters (BGCs), the RRE could theoretically be used as a bioinformatic handle to identify novel classes of RiPPs. In addition, due to the high affinity and specificity of most RRE-precursor peptide complexes, a thorough understanding of the RRE domain could be exploited for biotechnological applications. However, sequence divergence of RREs across RiPP classes has precluded automated identification based solely on sequence similarity. Here, we introduce RRE-Finder, a new tool for identifying RRE domains with high sensitivity. RRE-Finder can be used in precision mode to confidently identify RREs in a class-specific manner or in exploratory mode to assist in the discovery of novel RiPP classes. RRE-Finder operating in precision mode on the UniProtKB protein database retrieved ∼25,000 high-confidence RREs spanning all characterized RRE-dependent RiPP classes, as well as several yet-uncharacterized RiPP classes that require future experimental confirmation. Finally, RRE-Finder was used in precision mode to explore a possible evolutionary origin of the RRE domain. The results suggest RREs originated from a co-opted DNA-binding transcriptional regulator domain. Altogether, RRE-Finder provides a powerful new method to probe RiPP biosynthetic diversity and delivers a rich data set of RRE sequences that will provide a foundation for deeper biochemical studies into this intriguing and versatile protein domain.

**IMPORTANCE** Bioinformatics-powered discovery of novel ribosomal natural products (RiPPs) has historically been hindered by the lack of a common genetic feature across RiPP classes. Herein, we introduce RRE-Finder, a method for identifying RRE domains, which are present in a majority of prokaryotic RiPP biosynthetic gene clusters (BGCs). RRE-Finder identifies RRE domains 3,000 times faster than current methods, which rely on time-consuming secondary structure prediction. Depending on user goals, RRE-Finder can operate in precision mode to accurately identify RREs present in known RiPP classes or in exploratory mode to assist with novel RiPP discovery. Employing RRE-Finder on the UniProtKB database revealed several high-confidence RREs in novel RiPP-like clusters, suggesting that many new RiPP classes remain to be discovered.

## Abstract (precis)

Bioinformatics-powered discovery of novel ribosomal natural products (RiPPs) has historically been hindered by the lack of a common genetic feature across RiPP classes. Herein, we introduce RRE-Finder, a method for identifying RRE domains, which are present in a majority of prokaryotic RiPP biosynthetic gene clusters (BGCs). RRE-Finder identifies RRE domains 3,000 times faster than current methods, which rely on time-consuming secondary structure prediction. Depending on user goals, RRE-Finder can operate in precision mode to accurately identify RREs present in known RiPP classes or in exploratory mode to assist with novel RiPP discovery. Employing RRE-Finder on the UniProtKB database revealed several high-confidence RREs in novel RiPP-like clusters, suggesting that many new RiPP classes remain to be discovered.

## INTRODUCTION

**FIG 1:**
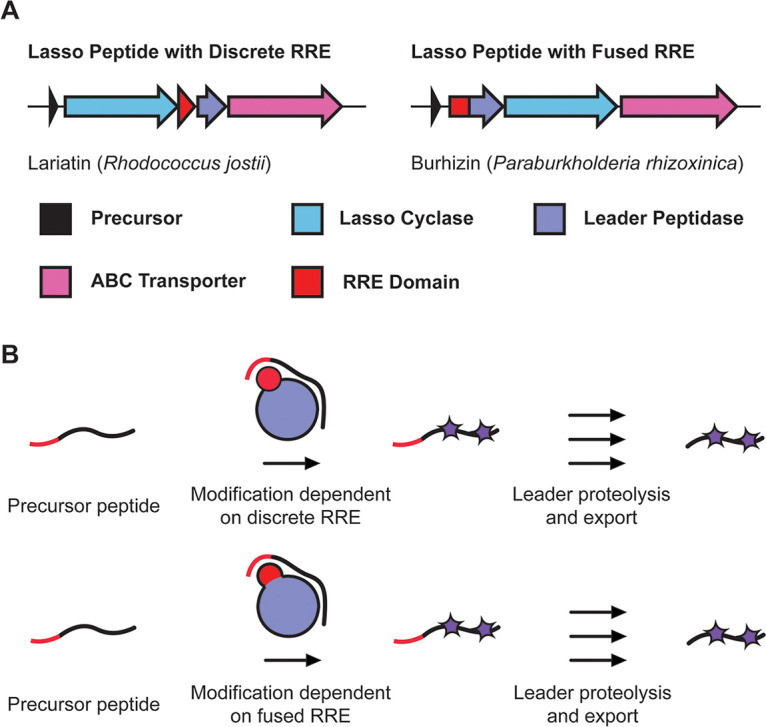
RRE-dependent RiPP biosynthesis. (A) RiPP BGCs encode one or more short precursor peptides; their genes often lie adjacent to those for the modifying enzymes, leader peptidases, and proteins for immunity and export (often ABC transporters). RRE domains are found as discrete polypeptides or fused to larger biosynthetic proteins. (B) Modifying proteins bind the leader region of the precursor peptide using RRE domains. Posttranslational modifications are then installed on the core region of the precursor peptide.

As of late 2019, nearly one-quarter of a million prokaryotic genomes were publicly available in the National Center for Biotechnology Information (NCBI) genome databases (1). This vast genomic resource has accelerated the pace of natural product discovery, with a recent surge of interest pertaining to the ribosomally synthesized and posttranslationally modified peptides (RiPPs) (2). RiPP biosynthesis starts with the ribosomal synthesis of a linear precursor peptide. The genes for RiPP precursor peptides are often short, hypervariable in sequence, and composed of two parts—an N-terminal leader region and a C-terminal core region. With a few notable exceptions, the precursor peptide is genetically encoded adjacent to one or more genes encoding proteins that bind with high specificity and affinity to the leader region of the precursor. This interaction facilitates subsequent posttranslational modification of the core residues (3). After modification is complete, the leader region is enzymatically removed and the mature RiPP product is exported from the producing organism (3) (Fig. 1). The exact nature of the posttranslational modifications is used to categorize RiPPs into individual classes, of which nearly 40 have been reported (2). For example, lanthionine linkages define the lanthipeptide class, while oxazol(in)e and thiazol(in)e heterocycles define the linear azol(in)e-containing peptide (LAP) class (4, 5).

Many RiPP biosynthetic proteins recognize and bind their cognate precursor peptide through a domain known as the RiPP recognition element (RRE) (6). The RRE consists of a conserved secondary structure of three N-terminal alpha helices followed by a three-stranded beta sheet. The precursor peptide binds in a cleft between the third alpha helix (α3) and the third beta strand (β3), forming an ordered, four-stranded, antiparallel beta sheet (Fig. S1). RRE domains can exist either as discretely encoded proteins (<100 residues) or as fusions to a larger protein domain (6–10). In cases where a RiPP biosynthetic gene cluster (BGC) encodes a discrete RRE protein, this protein binds the leader peptide and serves as a scaffold for recruiting the necessary modifying enzymes. All characterized RREs share structural similarity to PqqD, which is a protein involved in synthesis of pyrroloquinoline quinone (PQQ), a redox cofactor produced by many prokaryotes (11). Thus, the existence of a PqqD-like protein encoded near regulators, enzymes, and transporters is strongly indicative of an RRE-dependent RiPP BGC. The prevalence of PqqD-like proteins in RiPP BGCs led to the discovery of the RRE domain and its conservation across RiPP classes in 2015 (6). Before this, the importance of leader peptide recognition was established in the biosynthesis of a few RiPPs, such as nisin (lanthipeptide) and streptolysin S (LAP) (12, 13). In addition, an RRE-containing protein from microcin C7 biosynthesis (MccB) was cocrystallized with its cognate leader peptide in 2009, but owing to RRE sequence divergence, it was not appreciated at the time that other RiPP classes employ a similar domain (14).

10.1128/mSystems.00267-20.1 FIG S1 Structural homology and sequence divergence of the RRE. (A) The crystal structures of three RRE domains (excised for LynD and NisB) are shown from three RiPP classes. The leader peptide is highlighted in blue, while the conserved cleft in the RRE that binds the leader peptide (LP) is highlighted in green. (B) The truncated protein sequences of each RRE are shown, with the same regions responsible for substrate binding highlighted. (C) A sequence similarity network of the PqqD Pfam is shown. Sequences belonging to PF05402 (PqqD) are represented in the SSN. The network was generated at an alignment score of 25 (E value = 10^−25^) and is presented as a RepNode80 (protein sequences with greater than 80% identity are conflated to a single node). Nodes are colored gold if the gene co-occurs within two open reading frames of a radical SAM enzyme (i.e., a PqqE homolog), indicating that the protein may be a true PQQ biosynthesis protein. The vast majority of RRE-containing proteins are not retrieved by PF05402. The network was generated using EFI-EST (26) and visualized using Cytoscape (27). Download FIG S1, PDF file, 1.9 MB. Copyright © 2020 Kloosterman et al. 2020 Kloosterman et al. This content is distributed under the terms of the Creative Commons Attribution 4.0 International license.

Consistent with the rapid expansion of characterized RiPP BGCs, a diverse collection of modifications and enzymatic domains are found among the ∼40 known RiPP classes. However, the lack of a common genetic feature remains a major obstacle in the bioinformatic detection of novel RiPP classes. The fact that RRE domains are prevalent in prokaryotic RiPP BGCs provides an opportunity. Of the ∼30 known RiPP classes produced by prokaryotes, over 50% contain an identifiable RRE domain (Table S1). Considering that the RRE domain appears to be the most conserved class-independent feature in RiPP BGCs, it theoretically could be used as an imperfect but useful bioinformatic handle to expand known RiPP sequence-function space by identifying new RRE-dependent RiPP classes.

10.1128/mSystems.00267-20.8 TABLE S1 Prevalence of the RRE domain within RiPP classes. (A) RRE domains are present in over 50% of RiPP classes produced by prokaryotes. These classes are listed along with information pertaining to the type of RRE fusion present and the class-defining modification(s). The example product listed is an archetypal example of the class and not necessarily the first discovered member of this class. The year of BGC discovery refers to the year in which a product of this class was determined to be a RiPP. In cases where a BGC for more than one member of a class was discovered (e.g., thiopeptides), a representative literature citation is given. Note that ranthipeptides were reclassified as such in 2017, but these RiPPs had been previously bioinformatically characterized and classified as SCIFFs. LAP, linear azol(in)e-containing peptides; rSAM, radical *S*-adenosylmethionine. (B) Representative list of prokaryotic RiPP classes. The most populous RiPP classes produced by prokaryotes are listed, including whether these classes are predicted to be RRE dependent. Classes are listed as RRE dependent if at least one protein in the BGC is predicted to contain an RRE by RRE-Finder exploratory mode. Not all of these classes have been confirmed to be RRE dependent by experimental studies. Although there are no general trends as to which classes are RRE dependent, some enzymes—such as rSAM enzymes and cyclodehydratases—commonly co-occur with discrete or fused RRE domains. (C) Representative RRE domains that have been structurally characterized are listed. LAP, linear azol(in)e-containing peptides; PQQ, pyrroloquinoline quinone. Download Table S1, DOCX file, 0.03 MB. Copyright © 2020 Kloosterman et al. 2020 Kloosterman et al. This content is distributed under the terms of the Creative Commons Attribution 4.0 International license.

The strategy outlined above is complicated by the sequence diversity of the RRE domain (6, 9–11). For example, if a pairwise sequence alignment method (e.g., NCBI BLAST [15]) is used to compare RRE domains from two unrelated RiPP classes, sequence similarity will frequently not be detected, particularly in cases where the RRE domain is fused to a larger protein. The most appropriate Pfam (16) model (a family of proteins sharing sequence similarity) for defining the RRE domain is PF05402, which extensively covers bona fide PqqD proteins from PQQ-producing BGCs. PF05402 incompletely retrieves RRE-containing proteins from only a few other RiPP classes (e.g., lasso peptides and sactipeptides), and indeed, most RREs from other RiPP classes have no representation in this Pfam (17–19) (Fig. S1). These results underscore the inability of a single bioinformatic model to capture the breadth of RRE sequence diversity. Owing to the fact that RREs share considerable structural similarity, HHpred (20) is a more sensitive algorithm for detecting RRE domains. HHpred detects remote protein homology by aligning profile hidden Markov models (pHMMs; a model that defines amino acid frequency for a protein family) and comparing their (predicted) secondary structures. RREs were originally detected using this method by analyzing several RiPP-modifying enzymes, which showed consistent homology to PqqD (6). However, HHpred requires generation of a multiple sequence alignment (MSA) and secondary structure prediction using PSIPRED (21). These steps require several minutes of computing time per protein query, rendering the process unattractive for larger data sets and precluding global analyses of RRE diversity. In this work, we report a customized tool that permits the rapid and accurate detection of RREs in known and potentially novel RiPP classes with the principal goal of directing natural product hunters to the most fruitful areas of the RiPP sequence-function space.

## RESULTS AND DISCUSSION

### Development of RRE-Finder.

This work presents RRE-Finder, a new tool for mining RRE domains from microbial genomes. RRE-Finder has two modes of operation: The first is precision mode, which employs a set of 35 custom pHMMs designed to detect RRE domains in a class-dependent manner (Fig. S2) (see Data Set S1 at https://figshare.com/articles/Dataset_S1_MSA_files/12030624 and Data Set S2 at https://figshare.com/articles/Dataset_S2_HMM_files/12030651). The precision-mode pHMMs are primarily based on known RiPP classes—in most cases, representative RRE-containing proteins from these classes have been verified to bind their cognate precursor peptide through biophysical experiments, such as X-ray crystallography or fluorescence polarization binding assays. The second mode, exploratory mode, uses a truncated version of the HHpred (20) pipeline with a custom database of detected RREs. Depending on the end user’s objective, RRE-Finder can be used in precision mode to accurately predict the presence of an RRE domain as well as the likely RiPP class in which the precursor peptide belongs. Alternatively, in exploratory mode, the user can retrieve a wider array of putative RRE-containing proteins to assist in the discovery of novel RRE-dependent RiPP classes. RRE-Finder accelerates the process of identifying RRE domains by several orders of magnitude compared to HHpred. Precision mode, for instance, can analyze >5,000 protein sequences per second (Table S2A). In addition to 29 core models based on known RiPP classes, precision mode includes 6 auxiliary models based on high-confidence, novel RiPP classes. We justified the inclusion of these models based on repeated observation of RRE domains within RiPP-like genomic contexts across multiple prokaryotic species. The 35 pHMMs that comprise precision mode are provided in Data Set S2 (https://figshare.com/articles/Dataset_S2_HMM_files/12030651).

10.1128/mSystems.00267-20.2 FIG S2 Representative RiPP gene clusters for precision mode models. (A) One representative example is given for each RiPP class represented by one or more precision mode models. The 35 pHMMs comprising precision mode are provided in Data Set S2 (https://figshare.com/articles/Dataset_S2_HMM_files/12030651). The relevant class is shown in bold above the BGC, while the specific product encoded by the cluster is shown below the cluster. RRE domains are highlighted in red. In cases where RRE domains are fused to other domains, the red portion of the open reading frame represents the location of the RRE within the protein. QHNDH, quinohemoprotein amine dehydrogenase; DUF, domain of unknown function; rSAM, radical *S*-adenosylmethionine. (B) Description of RRE-containing proteins targeted by precision mode. In cases where one BGC contains more than one protein with an RRE, separate NCBI protein accession identifiers are given for each RRE. Download FIG S2, PDF file, 0.5 MB. Copyright © 2020 Kloosterman et al. 2020 Kloosterman et al. This content is distributed under the terms of the Creative Commons Attribution 4.0 International license.

10.1128/mSystems.00267-20.9 TABLE S2 RRE-Finder computing times and prediction accuracy. (A) RRE-Finder analysis times compared to HHpred. Both precision and exploratory modes of RRE-Finder significantly decrease analysis times compared to HHpred, the gold standard for detecting RREs. Exploratory mode has longer analysis times than precision mode, due to the detection of distant protein homology. However, exploratory mode is roughly 3,000 times faster than HHpred analysis. Analysis was carried out on an Intel Xeon E5-4640 at 2.4 GHz, using 4 threads. (B) Model validation of precision mode for select RiPP classes. Four populous classes of RiPPs were selected for thorough model validation, using the most recent published data sets of predicted BGCs for sactipeptides, ranthipeptides, lanthipeptides, and thiopeptides (24, 25, 32). These classes were chosen because of the high quality of the published data set used for comparison. In all cases, the proteins from each data set known to contain RRE domains were queried against the relevant precision model using hmmscan at tolerant, moderate, and stringent bit score cutoffs. To determine the false-positive rate of the lanthipeptide model, all LanB-type enzymes in the data set belonging to type II to IV lanthipeptide biosynthetic pathways were queried. These types of lanthipeptides are not predicted to contain RREs by detailed manual analysis and thus serve as a reasonable negative control. Because the number of class II to IV lanthipeptides is roughly twice that of class I lanthipeptides, the dataset of false positives was much larger for testing this model than for the other three models shown. To determine the false-positive rates of the sactipeptide, thiopeptide, and ranthipeptide models, a neighboring protein to each RRE domain was queried. The neighboring proteins queried were ABC transporters (for sactipeptides/ranthipeptides) and cyclodehydratase enzymes (for thiopeptides). Download Table S2, DOCX file, 0.02 MB. Copyright © 2020 Kloosterman et al. 2020 Kloosterman et al. This content is distributed under the terms of the Creative Commons Attribution 4.0 International license.

In general, for RiPP classes where an extensive survey of the bioinformatic space has been performed (e.g., lasso peptides [22, 23], sactipeptides and ranthipeptides [24], and thiopeptides [25]), custom pHMMs were built by first visualizing sequence space through use of a sequence similarity network (SSN) for all RRE-containing proteins in the data set (26). SSN visualization using Cytoscape (27) facilitated selection of the most diverse and nonredundant subset of RRE primary sequences for seed sequence alignment. In cases where a published data set was available for a given RiPP class, model prediction accuracy was gauged by using hmmscan (from the HMMER3 suite [28]) on the relevant data set using bit scores of 15, 25, and 35 (referred to here as tolerant, moderate, and stringent cutoffs). A given pHMM was considered acceptable if >95% of RRE-containing proteins within the data set were retrieved by the model at a bit score of 25 (Table S2B).

In cases where a deep bioinformatic profiling of a RiPP class had not been previously published or where a mature natural product is not known (i.e., clusters predicted by the auxiliary models), seed alignment input sequences were gathered using PSI-BLAST (29) to find diverse homologous sequences to a representative sequence from each given class. The generated pHMMs were considered valid if an hmmsearch of the UniProtKB database (30) with a bit score cutoff of 25 gave only hits within BGCs with architectures similar to those of the target class. In addition, characterized data sets of RiPP proteins (e.g., lanthipeptides [31, 32], lasso peptides [22, 23], and sactipeptides [24]) were used to test auxiliary models using hmmscan analysis. Models giving few or no hits were considered to have acceptably low false-positive rates.

Exploratory mode, on the other hand, was built for the detection of RRE domains with greater sequence divergence from those detected by precision mode. For this mode, we employed a variation of the HHpred pipeline to detect structural similarity to RRE domains. HHpred uses a clustered UniProt database (uniclust30) (33), which comprises a small, representative set of all UniProt protein sequence diversity. Query proteins are compared to the uniclust30 database to generate a representative protein family for the query, and the consensus sequence of this representative protein family is compared to those of other protein families. This search also incorporates comparison of (predicted) secondary structures. As such, HHpred can detect distantly related sequences and overlap in secondary structures between a query protein and the UniProt database. However, the vast search space used far exceeds what is necessary if the goal is to detect RRE domains.

To accelerate the HHpred pipeline for RRE detection, we first built a smaller, more specialized HHpred database, consisting of ∼2,400 diverse RRE sequences. These sequences were gathered by retrieving 5,000 RiPP BGCs from the antiSMASH database (34) using HHpred. Rather than manually curating the retrieved RREs in a class-specific manner, as was done for precision mode, all detected RREs were indiscriminately included. The only manual curation carried out was the removal of helix-turn-helix-containing proteins and other transcriptional regulators. While these proteins may display structural similarity to RREs, they are not involved in RiPP biosynthesis and therefore were excluded from the data set. The selected RREs were supplemented with 7 RREs from LAP BGCs and an RRE from a proteusin BGC, as no BGCs from these RiPP classes were present in the antiSMASH database.

**FIG 2:**
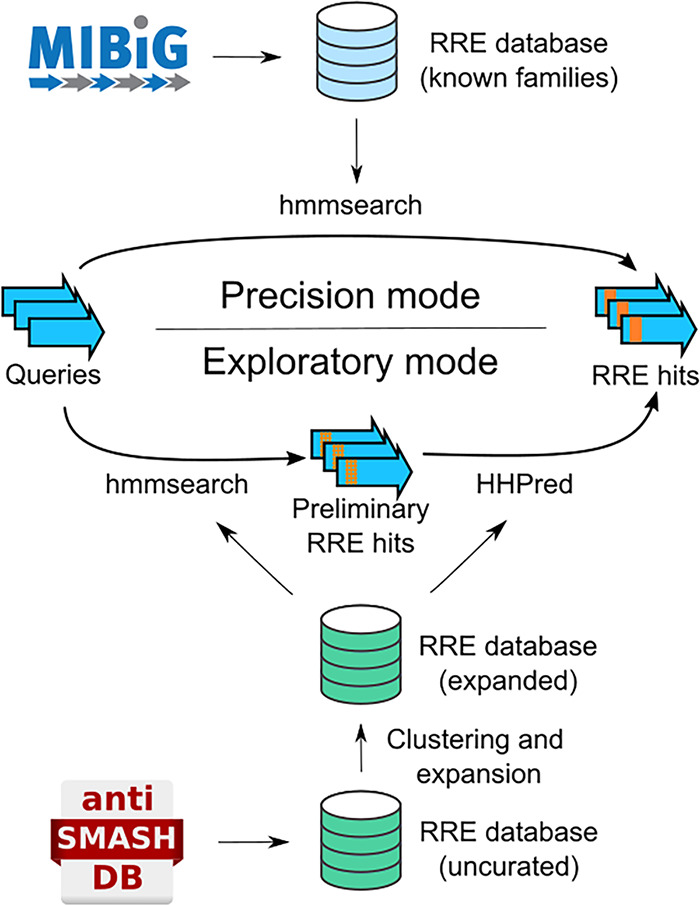
RRE-Finder employs two modes for RRE detection. Precision mode (top) uses a set of pHMMs to accurately predict RREs. These pHMMs are based on characterized RRE domains for individual RiPP classes, either from published data sets or from the MIBiG database. Exploratory mode uses a combination of pHMMs and a truncated HHpred pipeline (including secondary-structure prediction) to facilitate the identification of divergent RRE sequences (albeit with a higher false-positive rate).

The collection of ∼2,400 RREs was used to build databases for two filtering steps (Fig. 2). For the first filter, all RREs were clustered into representative protein families with MMSeqs2 (35), resulting in 377 RRE families. These RRE families were further enriched by querying each family against the uniclust30 database using HHblits, an iterative search tool from HHpred (36). For each of the 558 resulting RRE families, custom pHMMs were constructed, allowing an initial filtering step with hmmsearch (28). The second filtering step functions in a manner similar to that of HHpred. However, rather than using the uniclust30 database to retrieve a protein family for a query, we employed a smaller, custom HHpred database consisting of the ∼2,400 RRE sequences retrieved from the antiSMASH database and their related protein families retrieved by HHblits. When this custom database is used, only protein queries that are homologous to one of the 377 clustered RRE families will return results. For queries lacking homology, no protein family would be found in the database, effectively filtering out such sequences. Finally, exploratory mode compares the family of proteins homologous to a query protein to three RRE structures in the Protein Data Bank (PDB entries 5V1T, 5SXY, and 3G2B). Any proteins showing homology to these models are output as putative RRE domains. In all, by employing a small, custom library of RRE sequences, exploratory mode significantly accelerates detection of RREs relative to the standard HHpred pipeline.

### Model validation against the MIBiG database.

**FIG 3:**
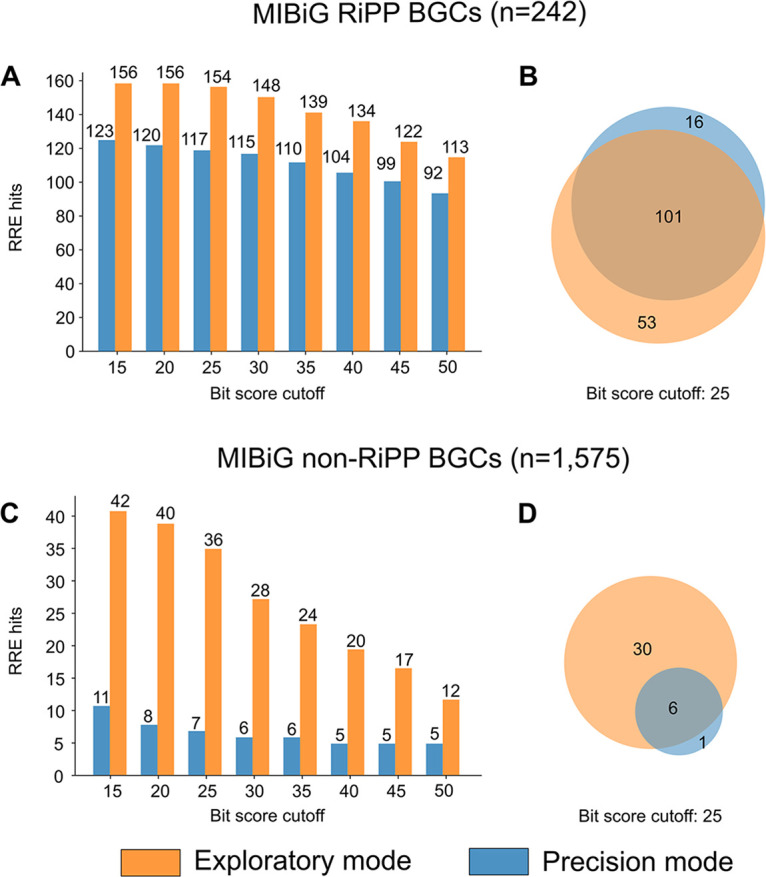
MIBiG validation of RRE-Finder. Both modes were used to retrieve RRE-containing proteins in 242 RiPP BGCs (A and B) and 1,575 non-RiPP BGCs (C and D) from the MIBiG database. With increasing bit score stringency, the number of RREs detected decreased in both types of BGCs (A and C). At a bit score of 25, exploratory mode of RRE-Finder detected most of the RREs found by precision mode in RiPP BGCs (B), as well as several other RREs. However, the number of RREs detected in non-RiPP BGCs was lower for precision mode than exploratory mode (D).

As an initial test of accuracy, RRE-Finder was evaluated in precision and exploratory modes against the MIBiG database (37). This database contains characterized BGCs for ∼2,000 natural products, including polyketides, nonribosomal peptides, and RiPPs. All proteins within the MIBiG set (version 1.4) of RiPP (*n* = 242) and non-RiPP BGCs (*n* = 1,575) were analyzed by RRE-Finder at tolerant, moderate, and stringent bit scores (Fig. 3).

In general, both precision and exploratory modes accurately predicted the presence of RRE domains in >90% of the RRE-dependent RiPP BGCs. Taken together, both modes retrieved 93% (115/122) of RRE-containing proteins found by HHpred (Table S3A). With increasing bit score stringency, the number of RRE sequences retrieved decreased in both RiPP and non-RiPP BGCs, as expected (Fig. 3). At all bit score cutoffs, exploratory mode predicted more RRE domains in RiPP BGCs (higher true-positive rate than precision mode), while precision mode retrieved fewer proteins from non-RiPP BGCs (lower false-positive rate than exploratory mode). After further analysis, we chose a bit score cutoff of 25 as a compromise between precision and recall. At this cutoff, most of the RREs found within the MIBiG set by precision mode were also found by exploratory mode (101/117) (Fig. 3). Only the RREs of linear azol(in)e-containing peptides (LAPs) (4) and streptides (38) proved more difficult to detect by exploratory mode (Table S3A). The inability of exploratory mode and HHpred to reliably predict LAP RRE domains may reflect a large diversity of leader peptide recognition sequences within this class that is better captured by the five distinct LAP models used by precision mode.

10.1128/mSystems.00267-20.10 TABLE S3 Validation of RRE-Finder modes against the MIBiG database. (A) RRE domains predicted by RRE-Finder and HHpred are grouped based on RiPP class. Precision and exploratory mode combined detect almost all of the RRE-containing proteins detected by HHpred (rightmost column). Precision mode readily detects RRE domains in known RiPP classes. Exploratory mode also detects these RREs but additionally retrieves putative RRE domains in thioviridamide-like and pheganomycin BGCs. Some of these RREs were also predicted by HHpred; thus, exploratory mode gives results in these cases similar to those obtained with HHpred. However, exploratory mode only sparingly detects RREs in the LAP and streptide RiPP classes. †, RRE domain experimentally determined to be nonfunctional and thus excluded from precision mode; *, 15 of these proteins show weak similarity to the ocin_ThiF_like domain (TIGR03693); **, RREs in radical SAMs encoded by bottromycin BGCs are typically detected by HHpred at a slightly lower probability than was used as the cutoff (∼70 to 90%). (B) Exploratory mode false-positives in non-RiPP BGCs. Exploratory mode retrieved a total of 51 proteins in non-RiPP BGCs at a bit score cutoff of 25. Many retrieved proteins were transcriptional regulators or proteins with a helix-turn-helix (HTH) motif, which is unsurprising considering structural homology to the RRE domain. Other false positives included several proteins with sequence homology to RRE-containing proteins in RiPP BGCs. Some BGCs in MIBiG have poorly defined boundaries and thus may contain genes from nearby natural product clusters. Thus, some false positives shown may be true RRE domains in adjacent RiPP clusters (e.g., MIBiG BGC0000696, the BGC for gentamicin, contains a neighboring LanB dehydratase and a LanC cyclase). Download Table S3, DOCX file, 0.02 MB. Copyright © 2020 Kloosterman et al. 2020 Kloosterman et al. This content is distributed under the terms of the Creative Commons Attribution 4.0 International license.

In contrast, precision mode detected only 66% (101/154) of the RREs retrieved by exploratory mode. A notable number (*n* = 17) of the RRE-containing proteins not detected by precision mode were those contained in LanB-like proteins, which are found in certain lanthipeptide and thiopeptide BGCs. It has been shown that the LanB RRE domain found in thiopeptide BGCs is possibly vestigial, as the cognate leader peptide is not required for catalytic processing (39). Exploratory mode also detected several (*n* = 14) RREs fused to dehydrogenase enzymes present in cyanobactin, LAP, and thiopeptide BGCs, which were not detected by precision mode. These RREs may also be vestigial; thus, precision mode does not include models for identifying these RRE-like domains. HHpred analysis similarly does not detect many of these potentially inactive RREs; thus, exploratory mode provides the best coverage of functional and vestigial RRE domains in this instance. We note that some of the RREs detected by exploratory mode, such as those from the thioamide-containing RiPP and pheganomycin pathways, are presumed to be functional but have yet to be experimentally validated (Table S3A).

While exploratory mode detects a greater number of RREs, it also displays a higher false-positive rate (e.g., proteins retrieved from known non-RiPP BGCs). The false positives primarily consisted of helix-turn-helix domains and proteins with homology to known RRE-containing proteins that occur in non-RiPP contexts, such as radical *S*-adenosylmethionine (rSAM) enzymes (Table S3B). Many DNA-binding regulators possess a helix-turn-helix domain, which are structurally homologous to RRE domains (Fig. S3A). Indeed, most RRE domains analyzed by HHpred show homology to known DNA-binding domains and regulatory elements (e.g., PDB entries 3DEE, 2G9W, and 2OBP). Because regulatory proteins are not known to bind or modify RiPP precursor peptides, RRE-Finder includes an option to filter results that correspond to such domains.

10.1128/mSystems.00267-20.3 FIG S3 Common RRE detection false positives. (A) Structural homology of the RRE to DNA-binding proteins. The RRE consists of a conserved secondary structure of three α-helices and three β-strands, highlighted in blue and red in the structures shown. This secondary structure is also present in many regulatory and DNA-binding elements, such as the truncated DNA-binding portion of the *Neisseria* protein shown. HHpred analysis also shows high structural similarity (>90% probability) between several DNA-binding elements and RRE-containing proteins. Sequence similarity between transcription regulators and RRE domains still remains low, with the two sequences shown sharing only 33% amino acid sequence identity. Thus, it is plausible that RRE domains evolved from transcriptional regulatory proteins. (B) RRE-containing proteins found in type II PKS clusters. The lymphostin BGC, a member of the pyrroloquinoline alkaloid class of RiPPs (57). Many pyrroloquinoline alkaloid (PQA) clusters contain both a PKS-NRPS module and one or more LanB-type enzymes containing internal RRE domains. (C) Structure of lymphostin, a RiPP derived from tryptophan. Download FIG S3, PDF file, 1.0 MB. Copyright © 2020 Kloosterman et al. 2020 Kloosterman et al. This content is distributed under the terms of the Creative Commons Attribution 4.0 International license.

RRE-Finder operating in either mode retrieved LanB-like proteins within polyketide BGCs. There is precedence for the assimilation of RiPP-modifying enzymes into polyketide pathways (31), although the RRE domain within these proteins may be vestigial (Fig. S3B). Thus, retrieval of proteins outside canonical RiPP BGCs may not always constitute a false positive. Further biochemical validation is required to confirm or refute a functional RRE in these instances.

Finally, some pHMMs employed by precision mode were generated largely using RRE sequences from the MIBiG database. In these cases, validation against MIBiG alone is not sufficient to confirm or refute whether these models exhibit appropriate recall and precision. As an orthogonal means of precision mode validation, we ran the hmmscan function for the ∼5,000 RiPP BGCs from the antiSMASH database used to generate the exploratory-mode database (34). As previously stated, these BGCs primarily belong to the lanthipeptide, thiopeptide, LAP, sactipeptide, and lasso peptide classes. Because this collection of BGCs includes RRE-dependent and RRE-independent RiPPs (e.g., class II to IV lanthipeptides) (40), there are BGCs anticipated to not be retrieved by precision mode. These clusters were purposely included in the analysis as a negative control. All proteins within the 5,000 BGCs were scanned by precision mode at tolerant, moderate, and stringent bit scores. The percentages of scanned BGCs predicted by precision mode to contain an RRE were 90%, 87%, and 83%, respectively. The 10% of BGCs not predicted to contain an RRE by precision mode were manually examined, with the majority belonging to RiPP classes that are RRE independent. Some BGCs also contained regulatory elements that represent false positives by HHpred; these proteins were appropriately not retrieved by precision mode. Thus, precision mode accurately predicts the presence of RREs in an unbiased collection of BGCs and appropriately omits RRE-independent RiPP clusters.

### Defining the scope of RRE-dependent RiPP BGCs.

**FIG 4:**
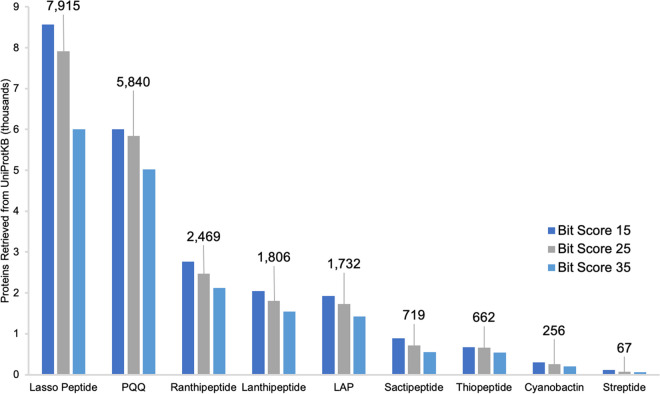
Summary of proteins retrieved from UniProtKB using precision mode. The numbers of proteins retrieved from the UniProtKB database are summarized for several classes of RiPPs. A scan of the entire UniProtKB database of nonredundant proteins was carried out at three bit scores. In cases where a given UniProt entry was retrieved by more than one precision model (due to partial model redundancy), the protein was counted only toward the model of higher significance. For classes with more than one precision-mode pHMM (e.g., LAPs and sactipeptides), the numbers presented are the sum of proteins retrieved by each individual model. Full data on proteins detected by each precision mode model are available in Data Set S3 (https://figshare.com/articles/Dataset_S3_RRE_domains/12568193). LAP, linear azol(in)e-containing peptide; PQQ, pyrroloquinoline quinone.

Next, we profiled the extent to which the RRE domain is present within sequenced genomes by mining the entire UniProtKB database (30). Using hmmsearch at a bit score threshold of 25, precision mode retrieved ∼25,000 proteins (∼13,000 nonredundant sequences) (Fig. 4). A parallel search using exploratory mode with regulators filtered out yielded ∼35,000 nonredundant RRE-containing proteins, almost completely encompassing the proteins retrieved by precision mode. As expected, the numbers of proteins retrieved by precision mode is larger than has been previously reported for virtually all RiPP classes, owing to on-going genome sequencing. For example, the thiopeptide precision model is the top-scoring model for more than 600 of the retrieved UniProtKB proteins, an ∼25% increase from the most recent bioinformatic survey of thiopeptide BGCs (25). In other cases, the number of retrieved proteins for a given model is misleading. For example, the precision mode model for discretely encoded lasso peptide RREs is the top-scoring model for almost 8,000 of the retrieved proteins. However, subsequent analysis revealed that only ∼4,000 of these sequences co-occur with the requisite leader peptidase and lasso cyclase. This number is more consistent with the most recent lasso peptide survey, which reported ∼3,000 lasso peptide BGCs (23, 41). Proteins retrieved by the discrete lasso peptide model often co-occur with other common RiPP enzymes, such as rSAM enzymes which represent ∼300 of the false positives. Thus, we caution that the number of proteins retrieved by any given model should not be equated to the number of BGCs specific to a particular RiPP class without analysis of the local genomic neighborhood. Full information on proteins retrieved by precision mode is available in Data Set S3 at https://figshare.com/articles/Dataset_S3_RRE_domains/12568193.

Figure 4 shows the number of retrieved proteins at tolerant, moderate, and stringent bit score cutoffs, as a measure of precision model specificity. Notably, due to partial model overlap in closely related RiPP classes (e.g., PQQs/lasso peptides and LAPs/thiopeptides/cyanobactins), the overall numbers of retrieved proteins for these models do not drastically increase going from moderate to tolerant bit scores. Thus, the majority of “false positives” detected by precision models at lower significance cutoffs represent an RRE-dependent RiPP BGC of a separate RiPP class. Notably, the only precision model that displayed a high count of real false positives, even at a bit score threshold of 15, was the discrete lasso peptide RRE model, for the reasons stated above.

**FIG 5:**
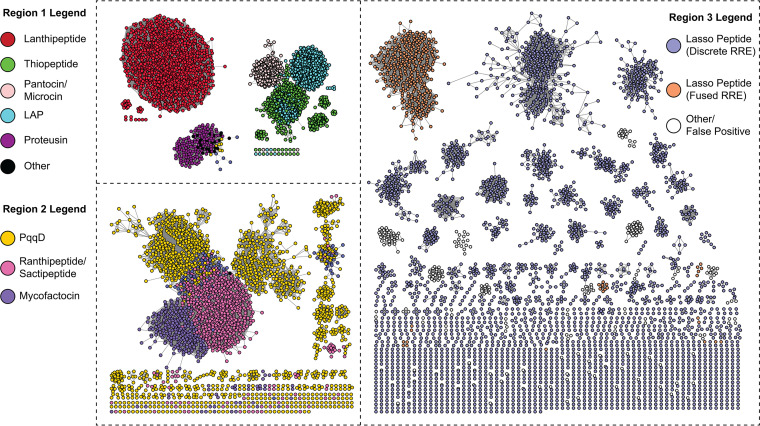
Sequence similarity network of UniProtKB proteins retrieved by precision mode. Shown is a RepNode60 SSN at an alignment score of 22 (sequences with >60% identity are conflated to a single node, and edges represent a BLAST expectation value better than 10^−22^). Proteins are colored based on the best-fit model by which they were detected. White nodes in region 3 represent proteins that were retrieved by the discrete lasso peptide RRE model but do not co-occur with the requisite leader peptidase and lasso cyclase. These proteins represent possible false positives from this model. The discrete lasso peptide RREs clustering with sactipeptides and ranthipeptides in region 2 are discretely encoded RRE proteins that co-occur with radical SAM enzymes. The SSN was generated using the Enzyme Similarity Tool (https://efi.igb.illinois.edu/efi-est/) (26).

The excised RREs from all proteins identified by precision mode were visualized using a sequence similarity network (SSN) (26). The SSN confirms known relationships between RREs in separate RiPP classes. For example, discretely encoded lasso peptide RREs (referred to as the B1 or E protein) group separately from RRE-leader peptidase fusions (known as the B2 or B protein), consistent with a different recognition sequence for these two varieties of lasso peptide (Fig. 5; Fig. S4A and B) (22, 23). In contrast, the heterocycloanthracins (LAPs) cluster more tightly with thiopeptides than other LAPs. This relationship was expected given that heterocycloanthracin and thiopeptide BGCs feature an RRE domain fused to an ocin-ThiF-like protein (TIGR03693) that delivers the peptide substrate to the biosynthetic enzymes (4, 42). In other LAP pathways, the RRE is fused to members of TIGR03882 (4, 6, 42, 43). Members of TIGR03882 recognize the peptide substrate through the RRE and perform cyclodehydration reactions, whereas these functions are carried out by separate proteins in thiopeptide and heterocycloanthracin clusters.

10.1128/mSystems.00267-20.4 FIG S4 Analysis of UniProtKB proteins retrieved by precision mode. (A) Sequence similarity network of retrieved UniProt proteins annotated by taxonomic origin. The UniProtKB database was searched using all precision models at a bit score cutoff of 25. All proteins retrieved are visualized on the sequence similarity network. The SSN is identical to Fig. 5 but has been recolored by taxonomy of the producing organism (alignment score of 22 [RepNode60]). The SSN was generated using EFI-EST (26) and visualized with Cytoscape (27). (B) The sequence similarity network in panel A was recolored according to the bit score significance of the match to a precision model (alignment score of 22 [RepNode60]). (C) Overlap of retrieved UniProt proteins in the most populous RiPP classes. Individual precision models for the most populous RiPP classes were employed for hmmsearch of the UniProtKB database at a bit score cutoff of 25. The total number of retrieved sequences for each model is in parentheses. The numbers within circles indicates model redundancy or overlap, owing to the same sequence being retrieved by more than one precision model at a bit score of 25. The discrete lasso peptide RRE model retrieves more proteins than anticipated because of similar motifs present in nonlasso BGCs. For example, many lasso peptide RREs co-occur in clusters with radical *S*-adenosylmethionine enzymes. In addition, there is significant overlap between the RREs of lasso peptides and those from PQQ clusters. Model overlap is similar to that shown at the more stringent bit score cutoff of 35. (D) Overlap of retrieved UniProt proteins in YcaO/RRE-dependent RiPP classes. Individual precision models for RiPP classes containing azole and azoline heterocycles were employed for hmmsearch of the UniProtKB database at a bit score cutoff of 25. The total number of retrieved sequences for each model is in parentheses, while numbers within circles indicate model redundancy or overlap, where the same protein sequence was retrieved by one or more precision models. Model overlap reveals that some numbers of retrieved proteins for precision mode are artificially high. For example, there are only ∼500 proteins retrieved by the thiopeptide model that co-occur with canonical thiopeptide modifying enzymes, such as the [4 + 2] cycloaddition enzyme. The other proteins retrieved by this model are heterocycloanthracins, which employ a highly similar leader peptide recognition sequence and RRE domain primary sequence. NHLP, nitrile hydratase-like leader peptide (58); HCA, heterocycloanthracin. Download FIG S4, PDF file, 2.4 MB. Copyright © 2020 Kloosterman et al. 2020 Kloosterman et al. This content is distributed under the terms of the Creative Commons Attribution 4.0 International license.

Another method to view RRE relatedness is through model redundancy (Fig. S4C and D). In cases where there is overlap in the proteins retrieved by multiple models, the redundancy is reflective of RREs in these classes binding their cognate leader peptides through similar sequence motifs. Similarly, lack of model overlap is indicative of a divergent leader peptide recognition sequence. For example, at a moderate bit score, there is virtually no overlap between the lanthipeptide-associated RRE domains with any other RiPP class, reflective of a unique recognition sequence not yet observed elsewhere (40, 44) (Fig. S4C). We note that model redundancy, particularly in RiPP BGCs with more than one RRE-containing protein, may suggest a similar recognition sequence on the cognate leader peptide. For example, the 3-thiaglutamate (pearlin RiPP class) BGC contains three proteins predicted to contain an RRE. The precision-mode pHMMs for these proteins display greater redundancy with each other than with any other model. This suggests comparable specificity of these RRE domains, as dictated by the α3 and β3 regions, and that these RREs likely bind the same region of the precursor peptide. However, this hypothesis will require further experimental evaluation.

### Evolution of the RRE domain.

Sequence similarity between recognition sequences in closely related RiPP classes suggests that the RRE domain emerged once and then diverged to recognize a variety of leader peptides. Because the leader peptide binds as an ordered beta-strand between the α3 helix and β3 strand of the RRE, substitutions of key α3 and β3 residues logically tune the RRE specificity toward the cognate peptide substrate. Analysis of residue-level conservation between RREs of divergent RiPP classes reveals that the α3 and β3 regions exhibit higher levels of residue conservation than the remainder of the domain, presumably due to selective pressure to conserve leader peptide-RRE contacts. This holds true even when closely related RiPP classes, such as LAPs and thiopeptides, are compared (Fig. S5A). The other regions of the RRE, which are not directly involved in leader binding, show lower levels of conservation.

10.1128/mSystems.00267-20.5 FIG S5 Evolution of the RRE domain. (A) Conservation of α3 and β3 regions of the RRE. Residue-level conservation was assessed using three metrics on eight precision mode models. The secondary structures principally responsible for binding the leader peptide (the α3 and β3 regions) were assessed separately from the remainder of the RRE domain. The region of the RRE with the greatest conservation per metric is indicated by red text. Individual RiPP classes were scored by selecting 10 divergent RREs from that class and excising the relevant substructure sequence. In some cases, pairs of RiPP classes that have significant mutual evolutionary relatedness were evaluated jointly; in these instances, a total of 20 sequences were used for the calculations (10 from each class). These data reveal a trend of higher conservation in the α3 and β3 regions of the RRE compared to other regions. Perhaps unsurprisingly, α3 displays the greatest conservation across RiPP classes, given that the contact with the leader peptide is primarily through side chain interactions as opposed to the β3 strand (primarily backbone interactions). HCA, heterocycloanthracin. (B) Representative phylogenetic tree for retrieved UniProt proteins. Shown are representative protein sequences retrieved by a hmmsearch of the UniProtKB database using precision mode. All proteins used for tree generation were truncated *in silico* to contain only the RRE domain. Arc segments are colored based on the precision mode model matched with the highest bit score. RRE domains from discrete lasso peptide BGCs share the most sequence similarity to non-RiPP regulatory proteins and thus branch most directly from the transcriptional regulator outgroup (appearing at ∼10 o’clock on the tree; PDB entry 3DEE). The tree was generated using FastTree (56) and visualized using the iTOL Web tool (59). Download FIG S5, PDF file, 0.8 MB. Copyright © 2020 Kloosterman et al. 2020 Kloosterman et al. This content is distributed under the terms of the Creative Commons Attribution 4.0 International license.

A representative phylogenetic tree of excised RRE domains retrieved by precision mode (bit score of 25) is consistent with the hypothesis that the RRE domain coevolved with the leader peptide to provide specificity in all RRE-dependent RiPP classes (Fig. S5B). The tree does not include all proteins retrieved by precision mode; rather, 10% of the proteins contained within each SSN cluster (Fig. 5) were included, along with all singletons, to generate a diversity-maximized collection of sequences spanning all RRE-dependent classes. The tree employs a helix-turn-helix DNA-binding protein as an outgroup (PDB entry 3DEE), as this protein scores well in HHpred searches of characterized RRE proteins, such as PqqD and LynD. As previously mentioned, it is plausible that the RRE domain evolved from DNA-binding regulatory elements, given the shared secondary structure and the similar function of these domains to specifically bind a stretch of DNA or a peptide (Fig. S3A). Unsurprisingly, the diversity-maximized tree shows a subset of the discrete lasso peptide RREs branching directly from the helix-turn-helix outgroup. Although discrete RREs called by this model are dispersed throughout the tree, the subset branching most directly from the outgroup is mostly representative of the false positives discussed previously (proteins not co-occurring with lasso peptide machinery). This may suggest that some of these false positives are DNA-binding proteins more closely related to true RREs (either in RiPP or non-RiPP contexts) and that discrete RREs evolved from these regulators. These proteins could also represent discrete RREs from currently uncharacterized RiPP classes. Furthermore, the tree shows clades of fused RRE domains branching off from discrete RREs as separate events for most RiPP classes. Some fused RRE types (e.g., fused lasso peptide RREs, ranthipeptides, and pantocins) form monophyletic clades branching from parent clades with discrete RREs. Other classes, like the lanthipeptides, are dispersed throughout many clades. This may indicate that fusion of the RRE domain to other domains occurred as separate events, even within some RiPP classes. These data are also consistent with the observed domain architectures, as some classes employ N-terminally fused RRE domains, while others exhibit C-terminal fusions (e.g., proteusins).

### Using RRE-Finder to identify novel RiPP clusters.

Theoretically, the sequence space retrieved by exploratory mode and the auxiliary models of precision mode encompasses RRE-containing proteins from yet-undiscovered RiPP classes. To explore this sequence space, divergent clusters mined from UniProtKB were manually examined for novel RiPP contexts. All proteins retrieved were grouped based on their best-fit Pfam model. Since we expected many regulatory elements or proteins with helix-turn-helix domains among the hits, we filtered these sequences after the first step of the exploratory pipeline, reducing the required computational time.

Among the remaining detected proteins, RRE-Finder reveals several potentially novel RiPP clusters with new gene architectures containing both discrete and fused RRE domains (Fig. S6). Included in these clusters are RRE-protein fusions that are not present in known classes, such as RRE-glycosyltransferase fusions and RRE-glutathione *S*-transferase fusions (Fig. S7). Of the nine potential RiPP BGCs shown in Fig. S7, four encode rSAM enzymes, which are found across several RiPP classes (24). The presence of rSAM enzymes in conjunction with predicted RREs is suggestive of a RiPP BGC. However, of the nine BGCs, only three contained probable precursor peptides (small genes of <150 amino acids, co-occurring with the RRE-containing protein), while four other BGCs contained precursor candidates predicted by RODEO. Therefore, manual curation of potentially novel BGCs found by RRE-Finder is strongly recommended. An overall sequence similarity network of the UniProtKB proteins accessed by exploratory mode is provided in Fig. S6A.

10.1128/mSystems.00267-20.6 FIG S6 RRE-containing proteins in UniProtKB found only by exploratory mode. (A) Sequence similarity network of retrieved UniProt proteins by exploratory mode. The UniProtKB database was searched using exploratory mode at a bit score cutoff of 25 (alignment score of 30 [RepNode60]). All proteins retrieved by exploratory mode, not inclusive of proteins retrieved by precision mode at the same bit score cutoff, are visualized on the sequence similarity network. Nodes are colored based on UniProt annotations that were highly represented in the network. Proteins that were retrieved by precision mode at bit score cutoffs under 25 have blue outlines. The network was generated using EFI-EST (26) and visualized with Cytoscape (27). (B) Proteins retrieved by RRE-Finder were grouped based on Pfam/TIGRFAM domain identification. The overlap with precision mode’s core models at a bit score threshold of 25 confirms that many known RRE fusions are detected by both modes, such as those containing YcaO and LanB dehydratase domains. Numbers of proteins retrieved by exploratory mode are inclusive of those retrieved by precision mode. Other novel RRE fusions are identified, such as fusions to metallo-β-lactamases, oxidoreductases, and glutathione *S*-transferases. RRE domains are also found in a number of unannotated small proteins, many of which are likely discrete RREs. Among the filtered proteins containing HTH domains (right column), the vast majority were annotated only as regulatory proteins. Notably, 1,869 short proteins (<120 residues) were filtered out during this step. Whether these proteins represent discrete RREs or simply small regulators could not be determined with the available data. Nevertheless, in most cases, no additional domain fusions were annotated among the filtered regulators. Download FIG S6, PDF file, 1.7 MB. Copyright © 2020 Kloosterman et al. 2020 Kloosterman et al. This content is distributed under the terms of the Creative Commons Attribution 4.0 International license.

10.1128/mSystems.00267-20.7 FIG S7 Example RiPP BGCs found by RRE-Finder. (A) Shown are nine BGCs that contain RRE domains in novel contexts. Proteins highlighted in red indicate proteins containing RRE domains as predicted by RRE-Finder. All RRE domain-containing proteins are listed in the accompanying table along with protein accessions. Some of the BGCs shown were mined using exploratory mode of RRE-Finder, while the others were mined using the auxiliary models of precision mode. In cases where a likely precursor peptide was predicted by RODEO but does not have an NCBI accession identifier, the putative precursor is annotated with an asterisk. (B) Description of RRE-containing proteins found by RRE-Finder. The letters used to identify a gene correspond to those used in the BGCs in panel A. Download FIG S7, PDF file, 0.5 MB. Copyright © 2020 Kloosterman et al. 2020 Kloosterman et al. This content is distributed under the terms of the Creative Commons Attribution 4.0 International license.

To date, almost no RiPP classes have been discovered using solely a bioinformatic approach. The mycofactocin class was initially predicted through a bioinformatic study on then-uncharacterized rSAM enzymes (45). In addition, the ranthipeptide class was defined solely using bioinformatics (as SCIFF [for “six cysteines in forty-five residues”] peptides) (46); however, this class was incorrectly assumed to be part of the existing sactipeptide class (24). In other cases, bioinformatics analyses have been used to expand diversity within known RiPP classes; for example, the streptide class has been expanded to include enzymes that diverge from the class-defining Lys-Trp cross-linking enzymes (38, 47). Also, one new RiPP class—the α-keto β-amino acid-containing peptides—and one RiPP-like class—the pearlins—were discovered through bioinformatic means (48, 49). These classes, however, were discovered through first identifying a divergent member of a known RiPP biosynthetic enzyme, rather than through a truly unbiased bioinformatic discovery. We expect that RRE-Finder will enable such discoveries.

### RRE-Finder incorporation into antiSMASH and RODEO.

To encourage the use of RRE-Finder, the algorithm has been made publicly available as a command-line tool (https://github.com/Alexamk/RREFinder). Protein queries can be supplied in FASTA or GenBank format. The tool is also capable of analyzing and updating antiSMASH and DeepBGC output files (50). Precision mode of RRE-Finder will be incorporated into the next release of antiSMASH. We further have incorporated the precision mode of RRE-Finder into RODEO (22), a genome-mining tool for RiPP discovery that provides genomic neighborhood visualization and prediction of precursor peptides. Protein-coding sequences within the genetic locus are annotated according to Pfam and TIGRFAM models to identify conserved domains and predict function. With the “include RRE scoring” function enabled, proteins with an identifiable RRE are annotated, along with their E-value significance. Both the command line version of RODEO (https://github.com/the-mitchell-lab/rodeo2) and the user-friendly Web tool version (http://rodeo.scs.illinois.edu) have been upgraded with the capabilities of RRE-Finder precision mode.

### Conclusion.

RRE-Finder rapidly and accurately detects RRE domains within known and potentially novel RiPP classes. Although not all RiPP classes are RRE dependent, the majority of prokaryotic RiPP classes are, including the largest known classes (i.e., class I lanthipeptides, lasso peptides, and ranthipeptides). RiPP natural products are a prime candidate for pathway engineering, as precursor peptides and their cognate modifying enzymes are all genetically encoded, typically within one BGC. However, efforts to bioinformatically predict RiPP BGCs lag behind those for predicting polyketide synthase (PKS) and nonribosomal peptide synthetase (NRPS) BGCs, due to a lack of strongly conserved protein domains spanning multiple RiPP classes. Through precision mode of RRE-Finder, we have shown that characterized RiPP classes contain more members than currently reported, although analysis of the genomic neighborhood should be performed to confirm class identity. Precision mode can further be employed, particularly with a tolerant bit score threshold, to predict novel RRE domains, such as those predicted by the auxiliary models. Finally, using RRE-Finder in exploratory mode reveals a set of ∼35,000 proteins that are predicted to contain an RRE, suggesting that additional classes of RRE-dependent RiPPs remain to be uncovered.

## MATERIALS AND METHODS

### Generation of precision mode models.

Precision mode was generated to accurately predict the presence of RRE domains specific to characterized RiPP classes, as well as RRE domains in selected bioinformatically predicted RRE-dependent RiPP clusters. There are 29 models employed by precision mode of RRE-Finder (not including auxiliary models), each specific to a given discrete or fused RRE protein within a characterized RiPP class (see Fig. S2 for represented classes). Each precision model consists of a custom profile hidden Markov model (pHMM). To build each pHMM, five to 20 representative sequences were selected from a given RRE class for seed sequence alignment. For several RiPP classes, an extensive bioinformatic survey of biosynthetic gene clusters has been conducted. When available, these data sets were employed to select seed sequences. The data sets included those describing known gene clusters for lanthipeptides (32), lasso peptides (22), thiopeptides (25), cyanobactins (51), bottromycins (52), linear azol(in)e-containing peptides (LAPs, including heterocycloanthracins, plantazolicins, nitrile hydratase-like leader peptides [NHLP]-derived RiPPs, Nif11-derived RiPPs, goadsporins, and cytolysins) (4), pantocins/microcins (53), and radical *S*-adenosylmethionine-derived RiPPs (including sactipeptides, ranthipeptides, quinohemoprotein amine dehydrogenases, and streptides). In these cases, sequence diversity was evaluated by generating a sequence similarity network (SSN) using the Enzyme Function Initiative Enzyme Similarity Tool (EFI-EST) (26) and visualizing the SSN with Cytoscape (27). Five to 20 sequences (depending on number of clusters in the SSN) were selected from divergent clusters on the SSN.

Bioinformatic data sets were not available for the following RRE-dependent RiPP classes: PQQ (11), proteusins, mycofactocins, trifolitoxins, α-keto β-amino acid-containing peptides, and pearlins. In these cases, a list of homologous sequences to a canonical gene were obtained with position iterative BLAST searching (PSI-BLAST) (29) with three iterations and an E-value cutoff of 0.05 in November 2019 using the GenBank nonredundant protein sequence database. Once a list of homologous sequences was obtained, an SSN was generated in the manner described above, and diverse sequences were selected for seed sequence alignment.

Seed sequences were analyzed for the presence of an RRE domain using the HHpred Web tool (https://toolkit.tuebingen.mpg.de) (20). A protein was considered to contain an RRE if part or all of the protein matched a PqqD model (either PDB entry 5SXY or 3G2B) with 80% probability or greater. All proteins containing RRE domains were excised *in silico* to contain only the residues matching the relevant PqqD model. Excised RRE sequences were then aligned using MAFFT 7.450 (54). MAFFT alignments were run using the L-INS-I alignment option. Multiple-sequence alignments were used directly to generate a pHMM using HMMER version 3.3 (28). Models were built using the hmmbuild function and pressed into binary form using the hmmpress function.

### Validation of precision mode models.

Precision mode models were validated against the full data sets from which seed sequences were chosen, excluding the sequences which were included in the pHMMs themselves. For each model, the pHMM was run against the full data set for the relevant RiPP class using the hmmscan function of HMMER3.3 (28). Hmmscan was run with a bit score cutoff of 25 and with all other options set to default. A given model was deemed functional if >95% of RRE-containing protein sequences in a data set were retrieved by the pHMM at this bit score threshold. In cases where this criterion was not met, sequences not retrieved by the model were used to enrich the original seed sequence alignment and an improved model was generated. In cases where an extensive bioinformatic survey was not available for a certain RiPP class, model accuracy was assessed in two ways: First, the set of homologous proteins generated by PSI-BLAST during model generation was tested against the pHMM using hmmscan with a bit score cutoff of 25. Second, an hmmsearch was performed using the HMMER3.3 Web tool (https://www.ebi.ac.uk/Tools/hmmer/search) against the UniProtKB database. The biosynthetic gene clusters surrounding gene hits were visualized using the RODEO Web tool (22) (http://rodeo.scs.illinois.edu). A model was considered valid if >95% of the proteins retrieved by PSI-BLAST were detected by the model and >90% of proteins retrieved from the UniProtKB database co-occurred with genes belonging to Pfams known to associate with that RiPP class. Finally, all models were tested for false-positive rates. All models were run against a data set of 3,000 protein sequences selected from across the data sets used for generating all precision mode models using hmmscan at a bit score cutoff of 35. Models were considered to have acceptably low false-positive rates if <100 hits for any given model belonged to a divergent RiPP class.

As described above, precision mode models were also validated against a set of ∼5,000 proteins from the antiSMASH database. These protein sequences were employed in the generation of exploratory mode and thus were a form of cross-validation between the two modes of RRE-Finder. This data set consists of RRE-containing proteins primarily from the thiopeptide, lasso peptide, lanthipeptide, sactipeptide, and LAP classes. Not all proteins contained within the data set canonically contain RRE domains, particularly those belonging to class II to IV lanthipeptides. All precision-mode models were assessed by hmmscan searches against this data set with bit score cutoffs of 15, 25, and 35 (representing tolerant, moderate, and stringent bit score thresholds).

### Generation of exploratory mode.

Exploratory mode was generated for the purpose of identifying RRE sequences with higher divergence from RREs in known RiPP classes in a more unbiased manner than precision mode. For exploratory mode, we constructed a truncated version of the HHpred pipeline (20). In this pipeline, a query sequence is first expanded with HHblits into a multiple sequence alignment (MSA) using a database of interest, in this case the uniclust30 database (36). The secondary structure of the MSA is predicted using the adds.pl script available in the PSIPRED function of the HHsuite tool (21). The MSA is then searched with HHsearch against a second database, which consists of three sequences from the Protein Databank (PDB) corresponding to RRE crystal structures (PDB entries 5V1T, 5SXY, and 3G2B). To closely mimic the HHpred pipeline, we used the uniclust30 database for MSA generation (version from August 2018 [https://uniclust.mmseqs.com]). This database contains all sequences from the UniProt database clustered with MMseqs2 (35) at a cutoff of 30% pairwise sequence identity.

For the initial generation of an RRE database, we used the above-mentioned pipeline to search 5,000 RiPP BGCs from the antiSMASH database against the uniclust30 database. Regions showing distant similarity to the reference RRE domains (probability, ≥40%; length, ≥50 residues) were extracted with 15 flanking residues on each side, and the extracted regions were resubmitted to the same pipeline with a higher cutoff to confirm the results (probability, ≥90%; length, ≥50 residues). Additional RRE sequences were added for the LAP, streptide, and proteusin RiPP families, for which no entries were available in the antiSMASH database.

The resulting database of RREs was used to generate a custom HHpred database as described in the documentation of the HHsuite tool, including the addition of secondary structure predictions with PSIPRED. In parallel, all RREs found were clustered with MMSeqs2 using default settings (pairwise identity, ≥80%) and the sequences in each cluster of RREs were aligned using MUSCLE (55). The resulting alignment was converted into .a3m format using the reformat.pl script available in the HHsuite tool. Each alignment was then further enriched with more homologous sequences from the UniProtKB database by using HHblits with the uniclust30 database with three iterations. Finally, the expanded alignments were converted into pHMMs using HMMER3.3.

In exploratory mode, each query is first subjected to hmmsearch using the pHMMs described above. Queries passing the initial cutoff (see main text) and with minimum alignment length of 50 residues have the relevant regions extracted, including 15 flanking residues on each side. The candidate RRE region is then subjected to the HHpred pipeline described above. In the first step of MSA generation, however, the custom database containing RRE regions is used instead of the uniclust30 database. RRE regions showing homology to the reference RRE domains (length, ≥50 residues; probability, ≥90%) are considered hits.

### Reducing false positives.

To remove sequences containing transcriptional regulators (a large source of false positives using exploratory mode), we constructed a list of Pfam pHMMs containing a variety of DNA-binding regulators and other helix-turn-helix domains that share structural homology to the RRE domain. Each resulting hit is searched against this database with hmmsearch using the trusted cutoffs of each pHMM. Overlap of a regulator with a retrieved RRE is indicated in the output file. Information on which Pfams were filtered out is available in Data Set S4 (https://figshare.com/articles/Dataset_S4_Pfam_filtering/12568136).

### Analysis of the MIBiG database.

The pipeline described above was used to analyze all proteins from the MIBiG database (version 1.4), using bit score cutoffs ranging from 15 to 50. The resulting hits were separated into those belonging to RiPP and non-RiPP BGCs. Hits from the RiPP BGCs were additionally clustered per RiPP class. RiPP BGCs containing only precursors were removed.

### Analysis of the UniProtKB database.

The pipeline described above was used to analyze all proteins from the UniProtKB/TrEMBL database (UniProt release 2019_09). A bit score cutoff of 25 was used for precision mode and the initial filter of exploratory mode. For exploratory mode, proteins identified as likely regulators were removed after the initial hmmsearch step in the exploratory pipeline.

For the discovery of new classes, UniProtKB hits found by both modes of RRE-Finder, in particular using the auxiliary models of precision mode, were annotated with Pfam models (version 32.0) (19). Several hits containing a Pfam domain that indicated an enzymatic activity were selected, and their genomic neighborhoods were investigated, as well as their overlap with antiSMASH gene clusters. In addition, the presence of RRE domains in these hits was confirmed by submitting to the HHpred Web tool (https://toolkit.tuebingen.mpg.de/tools/hhpred).

For analysis of the UniProtKB database using precision mode, the HMMER3.3 Web tools were used. Each model was individually run through hmmsearch of the UniProtKB database with a bit score cutoff of 25. Retrieved proteins for each model were compiled, and duplicate protein accessions were removed to determine the exact number of unique proteins detected by each precision model. Information on duplicate hits from two or more precision models were used to determine model overlap and RRE relatedness, as shown in Fig. S4.

### Generation of sequence similarity networks and a diversity-maximized phylogenetic tree.

The unique protein accessions from hmmsearch of the UniProtKB database using precision mode were directly used to generate an SSN using EFI-EST (26) (https://efi.igb.illinois.edu/efi-est/) and visualized with Cytoscape (27). All sequences were excised to consist of only the RRE domain using a custom script. This script employs hmmsearch to identify the residues of a protein corresponding to the query pHMM and includes only those residues in the FASTA output. All SSNs shown are either a RepNode60 or RepNode80 network, meaning that protein sequences sharing more than 60% or 80% sequence identity are conflated into one node on the network. In general, alignment scores for network visualization were chosen to reflect a cutoff where sequences with >40% sequence identity cluster together. For the networks shown in this work, these alignment scores were 22 and 25 (representative of E-value cutoffs of 10^−22^ and 10^−25^, respectively).

A diversity-maximized, maximum-likelihood phylogenetic tree was generated by first selecting a smaller subset of the sequences represented on the SSN. All sequences represented by clusters consisting of 1 to 3 nodes were included in the tree. For larger clusters, a random sampling of 10% of the sequences in the cluster was used for tree generation. All sequences were excised to contain only the RRE using the methods described above. The subset of sequences was used to generate a multiple-sequence alignment using MAFFT 7.450 (54). MAFFT alignments were run using the L-INS-I alignment option. The MSA was transformed into an approximate-maximum-likelihood tree using FastTree 2.1 (56) with the default Jones-Taylor-Thornton (JTT) model. The tree was visualized using the Interactive Tree of Life (iTOL) website (http://itol.embl.de/).

### Integration of RRE-Finder into RODEO and antiSMASH.

Precision mode models have also been incorporated into both the GitHub and Web tool versions of RODEO 2 (http://rodeo.scs.illinois.edu). Included is an option to score RRE domains, which, if selected, will show which precision-mode models are matched, along with the default Pfam matches. The integration of precision mode is in progress for version 6.0 of antiSMASH, which is currently in the development phase and will be reported elsewhere. In addition, the standalone RRE-Finder tool is available on GitHub (https://github.com/Alexamk/RREFinder) and is capable of detecting RREs in precision mode and exploratory mode directly from antiSMASH and DeepBGC output (50).
