# Supplementary material for: RRE-Finder: a Genome-Mining Tool for Class-Independent RiPP Discovery
Source: mSystems. 2020 Sep 1;5(5):e00267-20. doi: 10.1128/mSystems.00267-20 (PMC7470986; doi:10.1128/mSystems.00267-20)
Supplement: FIG S1 [file mSystems.00267-20-sf001.pdf]

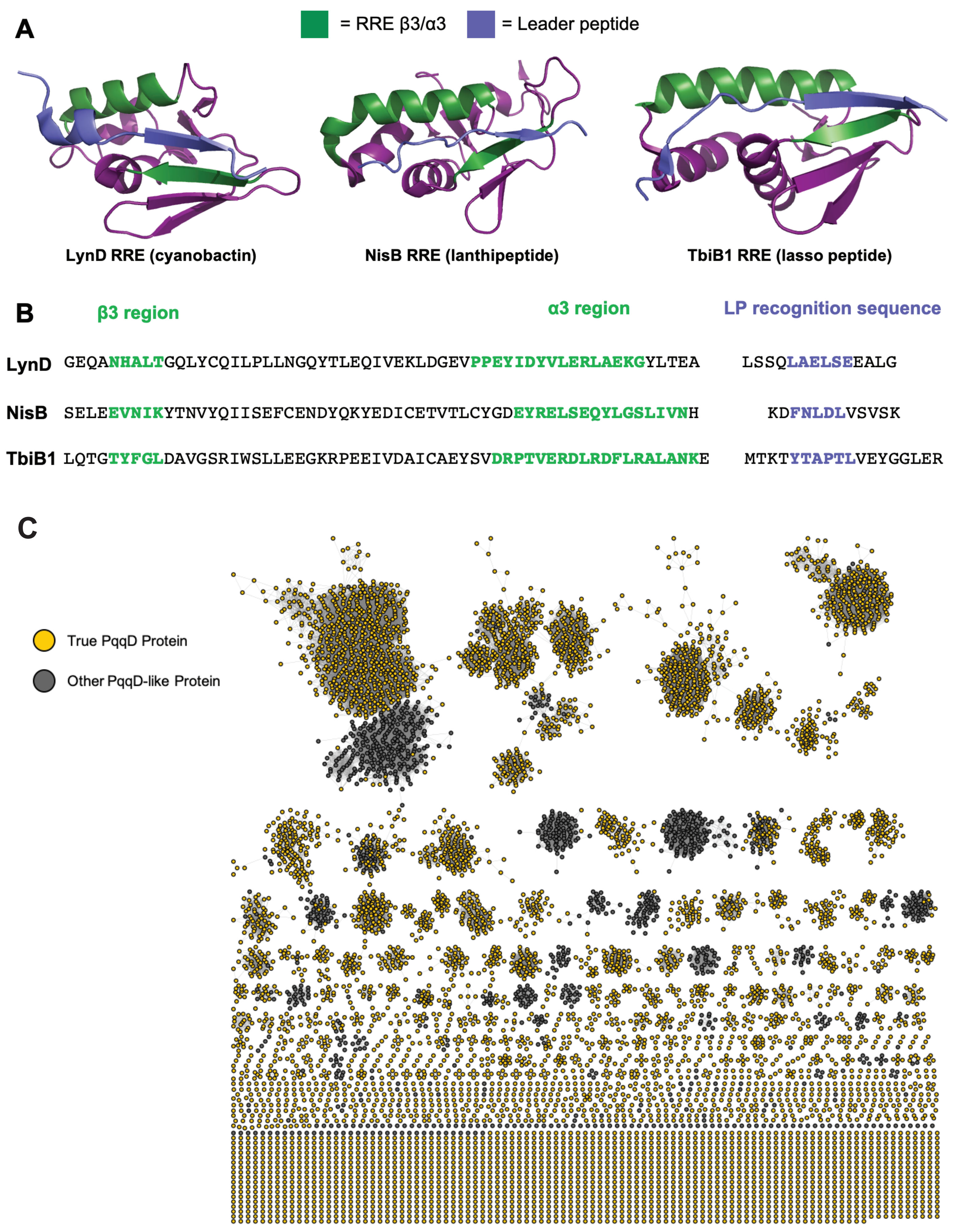
A
= RRE β3/α3
= Leader peptide
LynD RRE (cyanobactin)
NisB RRE (lanthipeptide)
TbiB1 RRE (lasso peptide)
B
β3 region
α3 region
LP recognition sequence
LynD GEQANHALTGQLYCQILPLLNGQYTLEQIVEKLDGEVPPEYIDYVLERLAEKGYLTEA LSSQLAELSEEALG
NisB SELEEVNIKYTNVYQIISEFCENDYQKYEDICETVTLCYGDEYRELSEQYLGSLIVNH KDFNLDLVSVSK
TbiB1 LQTGTYFGLDAVGSRIWSLLEEGKRPEEIVDAICAEYSVDRPTVERDLRDFLRALANKE MTKTYTAPTLVEYGGLER
C
True PqqD Protein
Other PqqD-like Protein
