# Supplementary material for: RRE-Finder: a Genome-Mining Tool for Class-Independent RiPP Discovery
Source: mSystems. 2020 Sep 1;5(5):e00267-20. doi: 10.1128/mSystems.00267-20 (PMC7470986; doi:10.1128/mSystems.00267-20)
Supplement: TABLE S1 [file mSystems.00267-20-st001.docx]

**A**

| **Class Name** | **Example Product** | **Class-Defining Modification and/or Enzyme** | **RRE Type** | **Taxonomic Distribution** | **Year of BGC Discovery** | **Citation DOI** |
| --- | --- | --- | --- | --- | --- | --- |
| Lanthipeptides | Nisin A | Lanthionines (Cys linkage to Ser/Thr β-carbon) | Fused to LanB protein (class I lanthipeptides only) | Archaea and bacteria | 1988 | [10.1038/333276a0](http://doi.org/10.1038/333276a0) |
| Pyrroloquinoline quinones (PQQ) | PQQ cofactor | Oxidative cyclization of Glu and Tyr, rSAM installed | Discrete RRE | Bacteria | 1989 | [10.1128/jb.171.1.447-455.1989](file:///Users/K_Smooth%201/Documents/RREFam%20Precision%20Models/10.1128/jb.171.1.447-455.1989) |
| Lasso peptides | Microcin J25 | Macrolactam ring with threaded lariat topology | Fused to leader peptidase or discrete | Archaea and bacteria | 1996 | [10.1128/jb.178.12.3661-3663.1996](https://dx.doi.org/10.1128%2Fjb.178.12.3661-3663.1996) |
| LAPs | Microcin B17 | Thiazol(in)es and (methyl)oxazol(in)es, YcaO-dependent | Fused to E1-like homolog (and sometimes YcaO as well) | Archaea and bacteria | 1996 | [10.1126/science.274.5290.1188](file:///Users/K_Smooth%201/Documents/RREFinder%20Final%20Drafts/10.1126/science.274.5290.1188) |
| Sactipeptides | Subtilosin | Sactionines (Cα linked thioether), rSAM installed | Fused to rSAM sactionine enzyme | Bacteria, primarily Firmicutes | 2000 | [10.1128/JB.182.11.3266-3273.2000](file:///Users/K_Smooth%201/Documents/RREFam%20Precision%20Models/10.1128/JB.182.11.3266-3273.2000) |
| Pantocins/  Microcins | Pantocin A | Claisen condensation of glutamic acid residues | Fused to E1-like homolog | Bacteria | 2003 | [10.1002/anie.200351054](http://doi.org/10.1002/anie.200351054) |
| Cyanobactins | Patellamide A | Protease with PatA homology | Fused to YcaO domain (azoline-containing cyanobactins only) | Cyanobacteria | 2008 | [10.1038/nchembio.84](http://doi.org/10.1038/nchembio.84) |
| Thiopeptides | Thiostrepton | [4+2] cycloaddition | Fused to the F-component of the cyclodehydratase | Bacteria, primarily Actinobacteria and Firmicutes | 2009 | [10.1073/pnas.0900008106](https://dx.doi.org/10.1073%2Fpnas.0900008106) |
| Mycofactocins | Mycofactocin | Crosslinking of Val and Tyr, rSAM installed | Discrete RRE | Actinobacteria (especially *Mycobacterium*) | 2011 | [10.1186/1471-2164-12-21](http://doi.org/10.1186/1471-2164-12-21) |
| Bottromycins | Bottromycin A1 | Macrolactamidine, YcaO-dependent | Fused to rSAM methyltransferase | Actinobacteria | 2012 | [10.1039/C2SC21190D](https://doi.org/10.1039/C2SC21190D) |
| Proteusins | Polytheonamide | Nitrile hydratase-derived leader peptide | Fused to rSAM epimerase and rSAM methyltransferase | Bacteria | 2012 | [10.1126/science.1226121](file:///Users/K_Smooth%201/Documents/RREFam%20Precision%20Models/10.1126/science.1226121) |
| Streptides | Streptide | Trp-Lys crosslinked, rSAM installed | Fused to rSAM enzyme | Mostly Firmicutes (especially *Streptococcus*) | 2015 | [10.1038/nchem.2237](http://doi.org/10.1038/nchem.2237) |
| Ranthipeptides | Freyrasin | Radical, non-Cα linked thioether, rSAM installed | Fused to rSAM enzyme | Bacteria, primarily Firmicutes | 2017 | [10.1021/jacs.9b01519](http://doi.org/10.1021/jacs.9b01519) |
| α-Keto β-amino acid-containing peptides | PlpA | Alpha keto amide linkages, rSAM installed | Discrete RRE | Archaea and bacteria | 2018 | [10.1126/science.aao0157](http://doi.org/10.1126/science.aao0157) |
| Rotapeptides | TQQ | Radical oxygen-to-alpha-carbon-linked peptides | Fused to rSAM enzyme | Bacteria, primarily Firmicutes | 2019 | [10.1021/jacs.9b05151](https://pubs.acs.org/doi/10.1021/jacs.9b05151) |
| Ryptides | RRR | Arg-Tyr crosslinked, rSAM installed | Fused to rSAM enzyme | Bacteria | 2019 | [10.1021/jacs.9b09210](https://pubs.acs.org/doi/full/10.1021/jacs.9b09210) |

**B**

| RiPP Class | Representative Example | Contains RRE | Reference DOI |
| --- | --- | --- | --- |
| Amidinotides | Pheganomycin | **Yes** | [doi.org/10.1038/nchembio.1697](http://doi.org/10.1101/338681) |
| Bottromycins | Bottromycin A1 | **Yes** | [doi.org/10.1021/jacs.7b09899](http://doi.org/10.1021/jacs.7b09899) |
| Cyanobactins | Patellamides | **Yes** | [doi.org/10.1046/j.1365-2958.2000.01982.x](https://doi.org/10.1046/j.1365-2958.2000.01982.x) |
| α-Keto β-amino acid-containing peptides | PlpA | **Yes** | [doi.org/10.1126/science.aao0157](http://doi.org/10.1126/science.aao0157) |
| Lanthipeptides | Nisin | **Yes** | [doi.org/10.1128/AEM.00635-15](http://doi.org/10.1128/AEM.00635-15) |
| Lasso peptides | Microcin J25 | **Yes** | [doi.org/10.1038/nchembio.2319](http://doi.org/10.1038/nchembio.2319) |
| Linear azol(in)e-containing peptides (LAPs) | Microcin B17 | **Yes** | [doi.org/10.1016/j.cbpa.2011.02.027](http://www.sciencedirect.com/science/article/pii/S1367593111000366) |
| Mycofactocin | Mycofactocin | **Yes** | [doi.org/10.1007/s00253-019-09684-4](http://doi.org/10.1007/s00253-019-09684-4) |
| Pantocins/Microcins | Pantocin A | **Yes** | [doi.org/10.1002/anie.200351053](http://doi.org/10.1002/anie.200351053) |
| Proteusins | Polytheonamide | **Yes** | [doi.org/10.1021/ja045749e](http://doi.org/10.1021/ja045749e) |
| Pyrroloquinoline quinones | PQQ | **Yes** | [doi.org/10.1073/pnas.0402640101](https://doi.org/10.1073/pnas.0402640101) |
| Ranthipeptides | Freyrasin | **Yes** | [doi.org/10.1021/jacs.9b01519](http://doi.org/10.1021/jacs.9b01519) |
| Rotapeptides | TQQ | **Yes** | [doi.org/10.1021/jacs.9b05151](https://pubs.acs.org/doi/10.1021/jacs.9b05151) |
| Ryptides | RRR | **Yes** | [doi.org/10.1021/jacs.9b09210](https://pubs.acs.org/doi/full/10.1021/jacs.9b09210) |
| Sactipeptides | Subtilosin | **Yes** | [doi.org/10.1016/j.cbpa.2013.06.031](http://doi.org/10.1016/j.cbpa.2013.06.031) |
| Streptides | Streptide | **Yes** | [doi.org/10.1038/nchem.2237](http://doi.org/10.1038/nchem.2237) |
| Pearlins | 3-Thiaglutamate | **Yes** | [doi.org/10.1101/338681](http://doi.org/10.1101/338681) |
| Thioamide-containing peptides | Thioviridamide | **Yes** | [doi.org/10.1021/acschembio.7b00677](http://doi.org/10.1021/acschembio.7b00677) |
| Thiopeptides | Thiostrepton | **Yes** | [doi.org/10.1021/jacs.8b03896](http://doi.org/10.1021/jacs.8b03896) |
| Atropitides | Tryptorubin | **No** | [doi.org/10.1021/jacs.7b06176](http://doi.org/10.1021/jacs.7b06176) |
| ComX | ComX186 | **No** | [doi.org/10.1038/nchembio709](http://doi.org/10.1038/nchembio709) |
| Crocagins | Crocagin A | **No** | [doi.org/10.1002/anie.201612640](http://doi.org/10.1002/anie.201612640) |
| Epipeptides | YydF | **No** | [doi.org/10.1038/nchem.2714](http://doi.org/10.1038/nchem.2714) |
| Glycocins | Sublancin 168 | **No** | [doi.org/10.1038/s41467-019-09065-5](http://doi.org/10.1038/s41467-019-09065-5) |
| Graspetides | Microviridin J | **No** | [doi.org/10.1021/ja00178a060](http://doi.org/10.1021/ja00178a060) |
| Linaridins | Cypemycin | **No** | [doi.org/10.1021/acschembio.7b00262](http://doi.org/10.1021/acschembio.7b00262) |
| Lipolanthines | Microvionin | **No** | [doi.org/10.1038/s41589-018-0068-6](https://doi.org/10.1038/s41589-018-0068-6) |
| Methanobactins | Methanobactin OB3b | **No** | [doi.org/10.1126/science.1098322](http://doi.org/10.1021/acschembio.7b00262) |
| Sulfatyrotides | RaxX | **No** | [doi.org/10.1073/pnas.1818275116](http://doi.org/10.1073/pnas.1818275116) |

**C**

| **Protein** | **RiPP Class** | **PDB Accession** | **UniProtKB Accession** | **Citation DOI** |
| --- | --- | --- | --- | --- |
| LynD | Cyanobactin | 4V1T | A0YXD2 | [10.1038/nchembio.1841](http://dx.doi.org/10.1038/nchembio.1841) |
| TruD | Cyanobactin | 4BS9 | B2KYG8 | [10.1002/anie.201306302](http://dx.doi.org/10.1002/anie.201306302) |
| NisB | Lanthipeptide | 5WD9 | P20103 | [10.1038/nature13888](http://dx.doi.org/10.1038/nature13888) |
| McbB | LAP | 6GOS | P23184 | [10.1016/j.molcel.2018.11.032](http://dx.doi.org/10.1016/j.molcel.2018.11.032) |
| TfuB1 | Lasso peptide | 6JX3 | Q47AT5 | [10.1021/acschembio.9b00348](http://dx.doi.org/10.1021/acschembio.9b00348) |
| TbiB1 | Lasso peptide | 5V1V | D1CIZ5 | [10.1073/pnas.1908364116](http://dx.doi.org/10.1073/pnas.1908364116) |
| MccB | Microcin | 6OM4 | Q47506 | [10.1039/c8sc03173h](http://dx.doi.org/10.1039/c8sc03173h) |
| PaaA | Pantocin | 5FF5 | Q9ZAR3 | [10.1021/jacs.5b13529](http://dx.doi.org/10.1021/jacs.5b13529) |
| PqqD | PQQ | 3G2B/5SXY | Q8P6M8 | [10.1002/prot.22461](http://dx.doi.org/10.1002/prot.22461) [10.1021/acs.biochem.7b00247](http://dx.doi.org/10.1021/acs.biochem.7b00247) |
| CteB | Ranthipeptide | 5WGG | A3DDW1 | [10.1021/jacs.7b01283](http://dx.doi.org/10.1021/jacs.7b01283) |
| SkfB | Sactipeptide | 6EFN | O31423 | [10.1074/jbc.RA118.005369](file:///Users/K_Smooth%201/Documents/RREFam%20Precision%20Models/10.1074/jbc.RA118.005369) |
| SuiB | Streptide | 5V1T | A0A0Z8EWX1 | [10.1073/pnas.1703663114](http://dx.doi.org/10.1073/pnas.1703663114) |
| TbtB | Thiopeptide | 6EC7 | D6Y502 | [10.1073/pnas.1905240116](http://dx.doi.org/10.1073/pnas.1905240116) |
