# Supplementary material for: RRE-Finder: a Genome-Mining Tool for Class-Independent RiPP Discovery
Source: mSystems. 2020 Sep 1;5(5):e00267-20. doi: 10.1128/mSystems.00267-20 (PMC7470986; doi:10.1128/mSystems.00267-20)
Supplement: FIG S2 [file mSystems.00267-20-sf002.pdf]

A

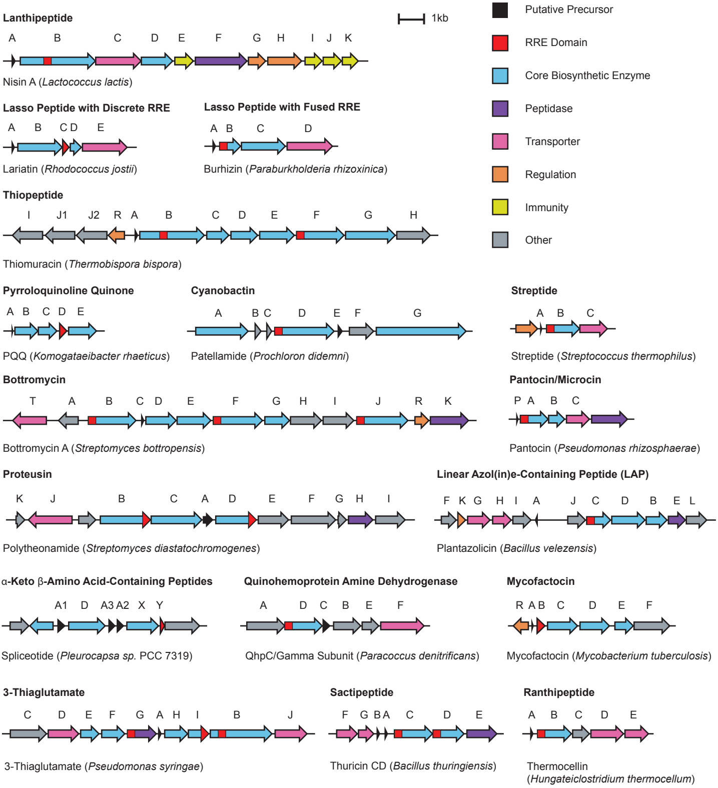

B

Natural Product	Protein	RRE Type	NCBI Accession
Nisin A	NisB	Fused to lanthipeptide dehydratase	ADJ56353.1
Lariatin	LarC	Discrete	BAL72548.1
Burhizin	BurB	Fused to lasso peptidase	CBW74825.1
Thiomuracin	TbtB	Fused to lanthipeptide dehydratase	ADG87277.1
Thiomuracin	TbtF	Fused to ocin-ThiF protein	ADG87281.1
PQQ	PqqD	Discrete	WP_034930240.1
Patellamide	PatD	Fused to cyclodehydratase	AAY21153.1
Bottromycin	BmbB	Fused to methyltransferase	CCM09442.1
Bottromycin	BmbF	Fused to methyltransferase	CCM09446.1
Bottromycin	BmbJ	Fused to methyltransferase	CCM09450.1
Polytheonamide	PoyB	Fused to methyltransferase	AFS60637.1
Polytheonamide	PoyD	Fused to epimerase	AFS60640.1
Plantazolicin	PznC	Fused to cyclodehydratase	CBJ61638.1
Thuricin CD	TrnC	Fused to rSAM enzyme	AED99784.1
Thuricin CD	TrnD	Fused to rSAM enzyme	AED99785.1
Streptide	SuiB	Fused to rSAM enzyme	ABJ66529.1
Spliceotide	PlpY	Discrete	WP_019503879.1
Pantocin	PaaA	Fused to ThiF protein	WP_043190265.1
Thermocellin	CteB	Fused to rSAM enzyme	WP_003517268.1
Mycofactocin	MftB	Discrete	WP_019735253.1
QHNDH	QhpD	Fused to rSAM enzyme	SDJ52620.1
3-Thiaglutamate	PmaB	Fused to short LanB enzyme	KPW26932.1
3-Thiaglutamate	PmaG	Fused to protease	KPW26903.1
3-Thiaglutamate	PmaI	Fused to DUF	KPW26921.1
