# Supplementary material for: RRE-Finder: a Genome-Mining Tool for Class-Independent RiPP Discovery
Source: mSystems. 2020 Sep 1;5(5):e00267-20. doi: 10.1128/mSystems.00267-20 (PMC7470986; doi:10.1128/mSystems.00267-20)
Supplement: TABLE S2 [file mSystems.00267-20-st002.docx]

**A**

| **Method** | **Dataset** | **Entries** | **Time Required (h)** |
| --- | --- | --- | --- |
| RRE-Finder (precision) | MIBiG (all) | 31,025 | 0.002 |
| RRE-Finder (exploratory) | MIBiG (all) | 31,025 | 0.2 |
| HHPred | MIBiG (RiPP only) | 2,513 | 54 |

**B**

| **Dataset** | **Bit Score** | | | | **Total in Dataset** | |
| --- | --- | --- | --- | --- | --- | --- |
|  | **15** | **25** | **35** |  | |  |
| Lanthipeptide, class I *(True-Positive)* | 1950 | 1910 | 1640 | 2020 | |  |
| Lanthipeptides, class II-IV *(False-Positive)* | 90 | 20 | 3 | 4453 | |  |
| Sactipeptide *(True-Positive)* | 799 | 769 | 690 | 865 | |  |
| Sactipeptide *(False-Positive)* | 1 | 1 | 0 | 865 | |  |
| Ranthipeptide *(True-Positive)* | 2241 | 2150 | 1960 | 2301 | |  |
| Ranthipeptide *(False-Positive)* | 10 | 7 | 4 | 2301 | |  |
| Thiopeptide ocin-ThiF Protein *(True- Positive)* | 495 | 492 | 440 | 515 | |  |
| Thiopeptide ocin-ThiF Protein *(False- Positive)* | 5 | 3 | 2 | 515 | |  |
