# Supplementary material for: RRE-Finder: a Genome-Mining Tool for Class-Independent RiPP Discovery
Source: mSystems. 2020 Sep 1;5(5):e00267-20. doi: 10.1128/mSystems.00267-20 (PMC7470986; doi:10.1128/mSystems.00267-20)
Supplement: TABLE S3 [file mSystems.00267-20-st003.docx]

**A**

| **RiPP Class** | **# curated BGCs** | **Protein annotation** | **Example MIBiG Accession** | **Example Protein** | **Total** | **Precision** | **Exploratory** | **HHPred** |
| --- | --- | --- | --- | --- | --- | --- | --- | --- |
| Lasso peptide | 35 | Leader peptidase | BGC0000581 | McjB | 12 | 8 | 10 | 7 |
|  | 35 | PqqD-like | BGC0000575 | LarC | 23 | 23 | 23 | 23 |
| Lanthipeptide, type I | 31 | LanB dehydratase | BGC0000535 | NisB | 30 | 29 | 30 | 27 |
|  | 31 | LanC-like | BGC001392 | PinC | 1 | 0 | 0 | 1 |
|  | 31 | MarR Regulator | BGC001392 | MarR | 1 | 0 | 0 | 1 |
| Thiopeptide | 24 | Dehydratase^†^ | BGC0000613 | TpdB | 17 | 0^†^ | 16 | 6 |
|  | 24 | Cyclodehydratase | BGC0000613 | TpdF | 2 | 2 | 2 | 2 |
|  | 24 | Radical SAM | BGC0001753 | TbtI | 1 | 0 | 1 | 1 |
|  | 24 | Hypothetical protein* | BGC0000603 | CltD | 21 | 19 | 17 | 17 |
|  | 24 | Dehydrogenase^†^ | BGC0000613 | TpdE | 5 | 0^†^ | 4 | 3 |
| Cyanobactin | 13 | Cyclodehydratase | BGC0000475 | PatD | 8 | 8 | 8 | 8 |
|  | 13 | Dehydrogenase^†^ | BGC0000475 | PatG | 8 | 0^†^ | 8 | 8 |
| LAP | 10 | Cyclodehydratase | BGC0000569 | PtnD | 8 | 8 | 1 | 3 |
|  | 10 | Dehydrogenase^†^ | BGC0000565 | GodE | 2 | 0^†^ | 2 | 2 |
|  | 10 | Hypothetical protein | BGC0000567 | TfxC | 1 | 1 | 1 | 1 |
| Thioamide-containing peptide (thioviridamide) | 3 | Methyltransferase | BGC0000625 | TvaG | 3 | 0 | 3 | 1 |
| Sactipeptide | 4 | Radical SAM | BGC0000600 | ThnB | 5 | 4 | 3 | 4 |
| Bottromycin | 4 | Radical SAM | BGC0000468 | BmbB | 12 | 12 | 12 | 0** |
| Pheganomycin | 1 | Radical SAM | BGC0001148 | Pgm3 | 1 | 0 | 1 | 1 |
| Proteusin | 1 | Radical SAM | BGC0000598 | PoyB | 1 | 1 | 1 | 1 |
|  | 1 | Radical SAM | BGC0000598 | PoyC | 1 | 1 | 1 | 1 |
|  | 1 | Radical SAM | BGC0000598 | PoyD | 1 | 1 | 1 | 1 |
| α-Keto β-amino acid-containing peptide | 1 | Radical SAM | BGC0001745 | PlpD | 1 | 1 | 1 | 1 |
| Streptide | 1 | Radical SAM | BGC0001209 | SuiB | 1 | 1 | 0 | 0 |
| Microcin | 1 | ThiF-like | BGC0000585 | MccB | 1 | 1 | 1 | 1 |
| Pearlin (3-thiaglutamate) | 1 | LanB dehydratase | BGC0001486 | PmaJ | 1 | 1 | 1 | 0 |
|  | 1 | Hypothetical protein | BGC0001486 | PmaI | 1 | 1 | 1 | 1 |
|  | 1 | Peptidase | BGC0001486 | PmaG | 1 | 1 | 1 | 1 |

**B**

| False-Positive Type | Number of Proteins Retrieved |
| --- | --- |
| Transcription Regulators/HTH Domains | 8 |
| Associated with Known RiPPs | 17 |
| Other | 11 |
