# Supplementary material for: RRE-Finder: a Genome-Mining Tool for Class-Independent RiPP Discovery
Source: mSystems. 2020 Sep 1;5(5):e00267-20. doi: 10.1128/mSystems.00267-20 (PMC7470986; doi:10.1128/mSystems.00267-20)
Supplement: FIG S3 [file mSystems.00267-20-sf003.pdf]

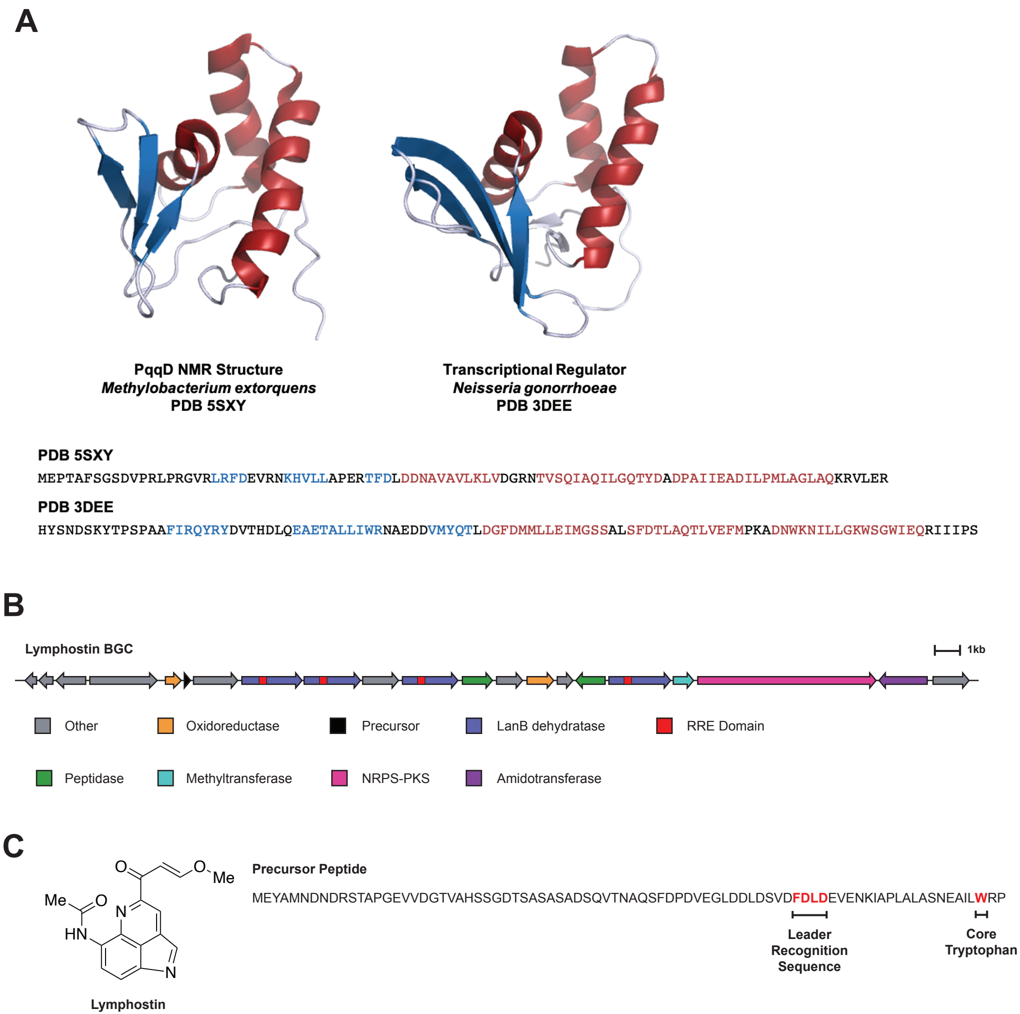

A
PqqD NMR Structure
Methylobacterium extorquens
PDB 5SXY
Transcriptional Regulator
Neisseria gonorrhoeae
PDB 3DEE
PDB 5SXY
MEPTAFSGSDVPRLPRGVRLRFDEVRNKHVLLAPERTFDLDDNAVAVLKLVDGRNTVSQIAQILGQTYDADPAIIEADILPMLAGLAQKRVLER
PDB 3DEE
HYSNDSKYTPSPAAFIRQYRYDVTHDLQEAETALLIWRNAEDDVMYQTLDGFDMMLLEIMGSSALSFDTLAQTLVEFMPKADNWKNILLGKWSGWIEQRIIIPS
B
Lymphostin BGC
1kb
Other
Oxidoreductase
Precursor
LanB dehydratase
RRE Domain
Peptidase
Methyltransferase
NRPS-PKS
Amidotransferase
C
O
O
Me
Me
O
N
HN
N
Lymphostin
Precursor Peptide
MEYAMNDNDRSTAPGEVVDGTVAHSSGDTSASASADSQVTNAQSFDPDVEGLDDLDSVDFDLDEVENKIAPLALASNEAILWRP
Leader Recognition Sequence
Core Tryptophan
