# Supplementary material for: RRE-Finder: a Genome-Mining Tool for Class-Independent RiPP Discovery
Source: mSystems. 2020 Sep 1;5(5):e00267-20. doi: 10.1128/mSystems.00267-20 (PMC7470986; doi:10.1128/mSystems.00267-20)
Supplement: FIG S4 [file mSystems.00267-20-sf004.pdf]

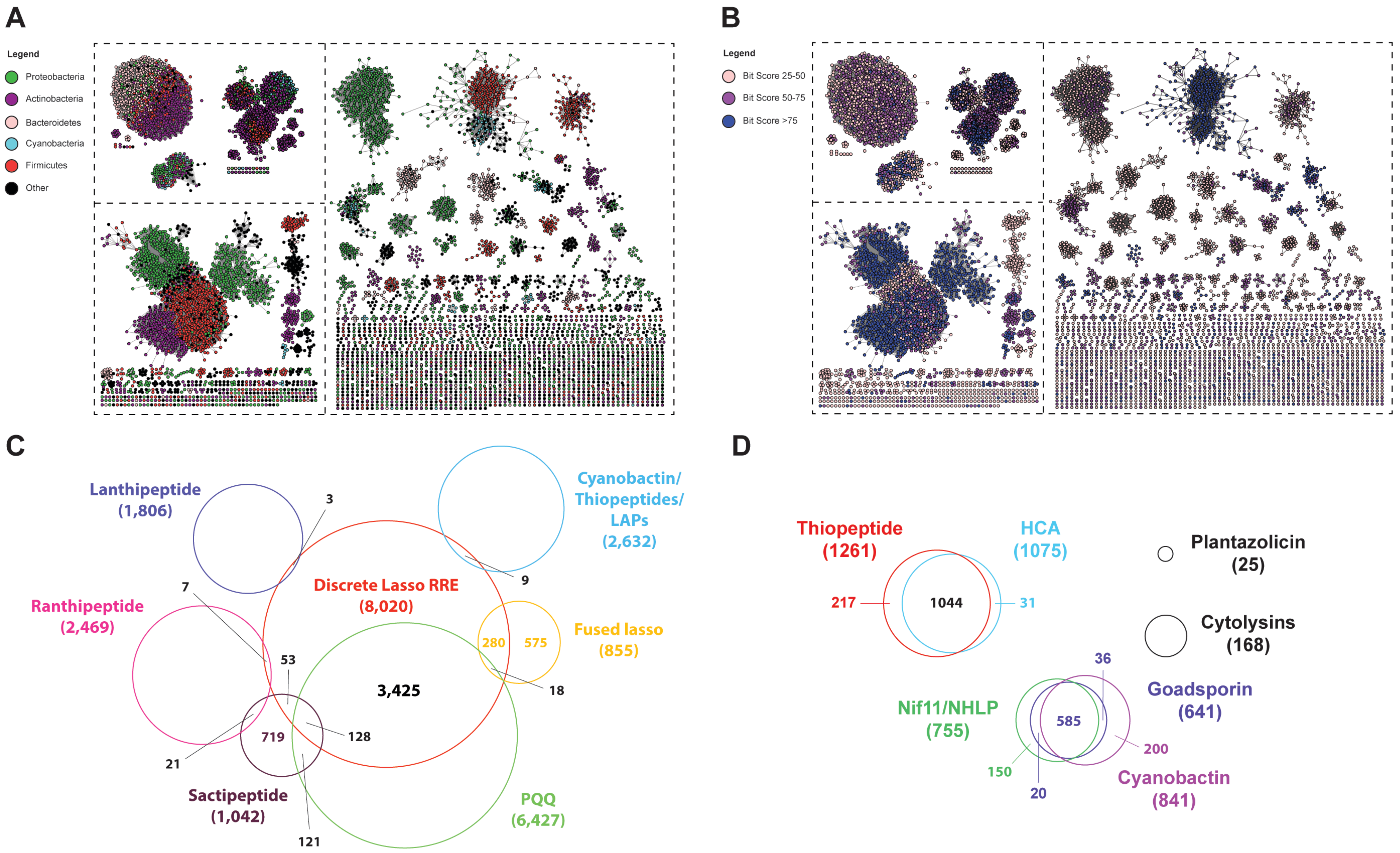

A
Legend
Proteobacteria
Actinobacteria
Bacteroidetes
Cyanobacteria
Firmicutes
Other
B
Legend
Bit Score 25-50
Bit Score 50-75
Bit Score >75
C
Lanthipeptide (1,806)
3
Cyanobactin/ Thiopeptides/ LAPs (2,632)
9
7
Discrete Lasso RRE (8,020)
Ranthipeptide (2,469)
280
575
Fused lasso (855)
53
3,425
18
719
128
21
Sactipeptide (1,042)
PQQ (6,427)
121
D
Thiopeptide (1261)
HCA (1075)
Plantazolicin (25)
217
1044
31
Cytolysins (168)
36
Goadsporin (641)
Nif11/NHLP (755)
585
200
150
20
Cyanobactin (841)
