# Supplementary material for: RRE-Finder: a Genome-Mining Tool for Class-Independent RiPP Discovery
Source: mSystems. 2020 Sep 1;5(5):e00267-20. doi: 10.1128/mSystems.00267-20 (PMC7470986; doi:10.1128/mSystems.00267-20)

A

	Shannon Information Entropy			ConSurf (0-9 scale)			AACon (0-9 scale)		
	α3 helix	β3 strand	other	α3 helix	β3 strand	other	α3 helix	β3 strand	other
Goadsporin	**0.81**	0.65	0.45	**7**	6	4	**7**	6	4
Cyanobactin	**0.75**	0.59	0.39	**7**	6	3	**7**	6	4
Goadsporin and Cyanobactin	**0.62**	0.54	0.21	**6**	**6**	2	**6**	5	2
Discrete Lasso peptide	**0.43**	0.33	0.23	**4**	3	2	**4**	3	2
Fused Lasso peptide	**0.51**	0.32	0.31	**5**	3	3	**4**	3	2
Discrete and Fused Lasso peptide	**0.27**	0.22	0.13	**3**	**3**	1	**3**	2	1
Thiopeptide	**0.76**	0.72	0.56	**7**	**7**	6	**7**	**7**	5
HCA	**0.82**	0.74	0.58	**8**	7	6	**8**	7	5
Thiopeptide and HCA	**0.71**	0.64	0.49	**7**	6	5	**7**	6	5
Ranthipeptide	**0.68**	0.57	0.42	**7**	6	4	**7**	5	4
QhpD	**0.71**	0.59	0.47	**7**	6	5	**7**	6	5
Ranthipeptide and QhpD	**0.54**	0.43	0.36	**5**	4	4	**5**	4	3

B

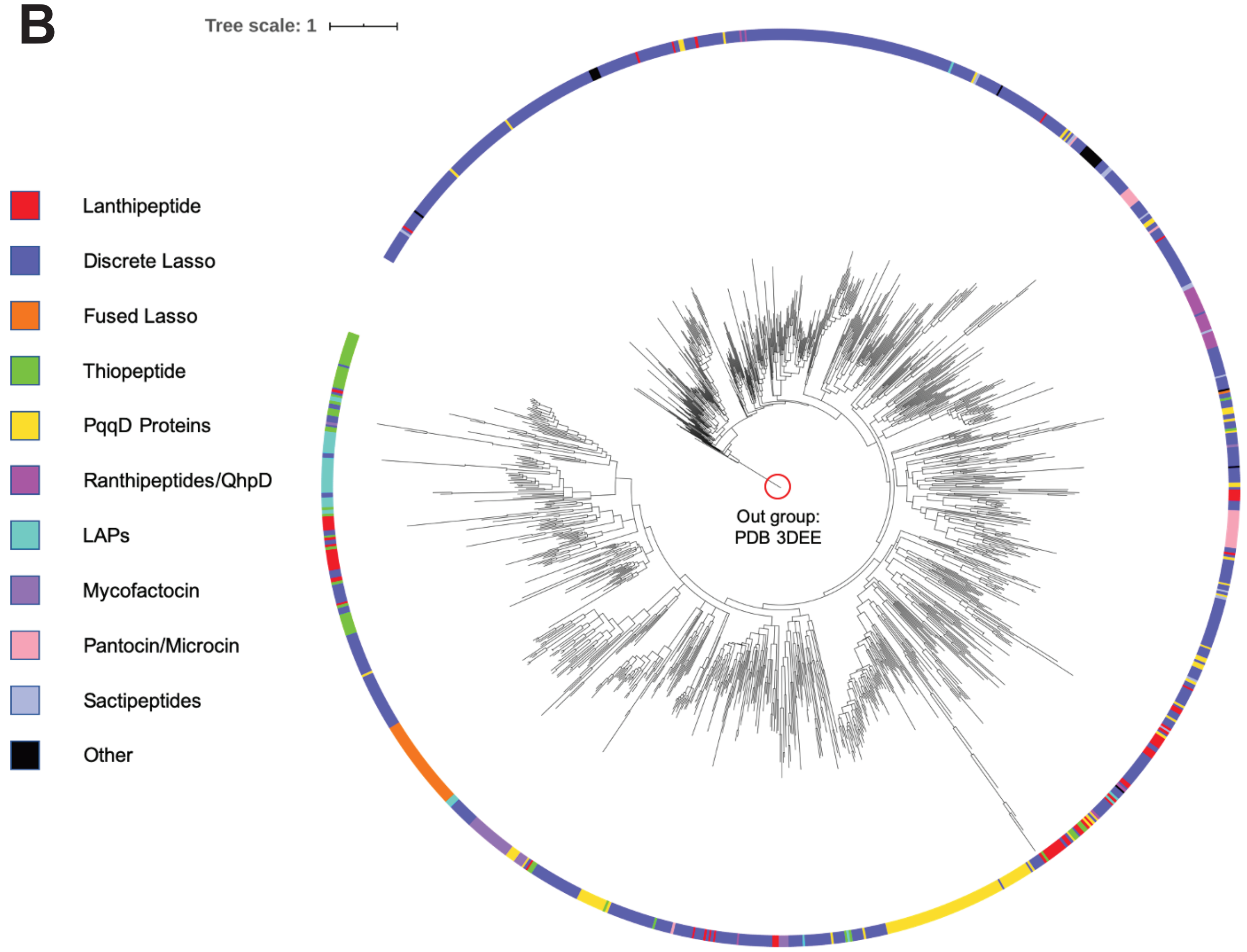

Supplement: FIG S5 [file mSystems.00267-20-sf005.pdf]
