# Supplementary material for: RRE-Finder: a Genome-Mining Tool for Class-Independent RiPP Discovery
Source: mSystems. 2020 Sep 1;5(5):e00267-20. doi: 10.1128/mSystems.00267-20 (PMC7470986; doi:10.1128/mSystems.00267-20)
Supplement: FIG S6 [file mSystems.00267-20-sf006.pdf]

A

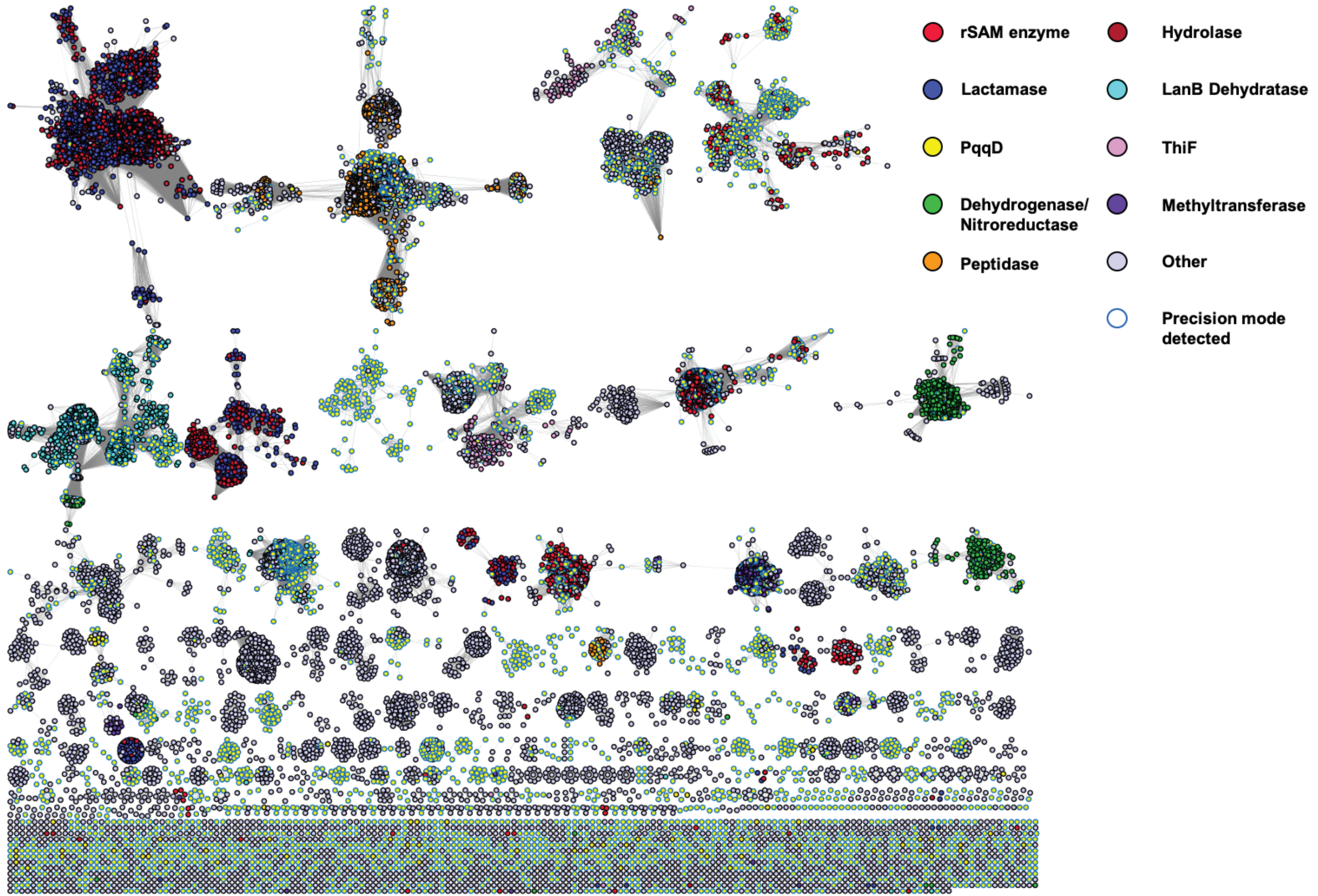

B

Protein domain annotation	Proteins found by exploratory mode after filtering	Proteins found by precision mode core models	Proteins eliminated by regulatory Pfam filtering
DNA-binding proteins and/or regulators	0	0	22.357
Other (length ≥ 120 amino acids)	16,595	1,094	20,267
Short proteins (length < 120 amino acids)	3,341	952	1,869
Metallo-β-lactamase	11,320	1	7
PqqD	10,994	9,128	18
Radical SAMs and/or Fe-S-binding domains	3,919	2,491	0
LanB dehydratase	3,313	1,888	2
Nitroreductase	1,039	10	4
YcaO protein	919	837	0
Methyltransferases	813	11	65
Transglutaminase	644	552	0
Ocin-ThiF-like	589	566	0
Memo proteins	463	5	0
Oxidoreductase	104	0	0
Tryptophan halogenase	75	0	0
Cyclic nucleotide binding domain	67	4	19
Tetratricopeptide repeat	66	4	18
Peptidase	64	2	81
Glycosyltransferase	56	20	1
Asparagine synthetase	19	2	0
Cupin domain	18	0	0
LanC cyclase	13	0	0
Glutathione-*S*-transferase	10	0	0
Carbamoyltransferase	8	0	0
