# Supplementary material for: RRE-Finder: a Genome-Mining Tool for Class-Independent RiPP Discovery
Source: mSystems. 2020 Sep 1;5(5):e00267-20. doi: 10.1128/mSystems.00267-20 (PMC7470986; doi:10.1128/mSystems.00267-20)
Supplement: FIG S7 [file mSystems.00267-20-sf007.pdf]

A

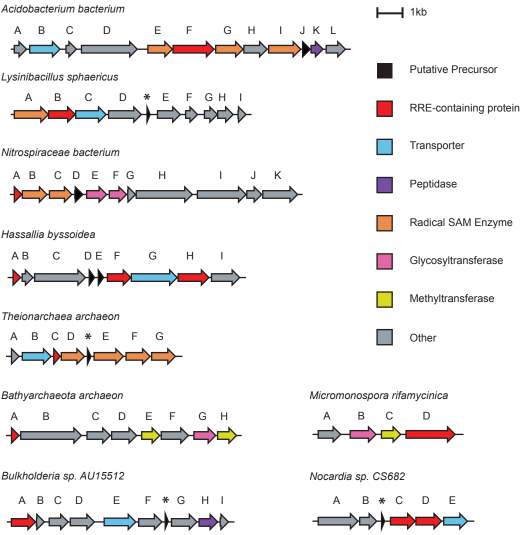

B

Organism	Gene	RRE Type	NCBI Accession
Acidobacterium bacterium	F	Fused to tetratricopeptide domain	OFW29522.1
Lysinibacillus sphaericus	B	Fused to glutathione *S*-transferase	WP_069508305.1
Nitrospiraceae bacterium	A	Discrete	RPI38387.1
Hassallia byssoidea	A	Discrete	KIF30015.1
Hassallia byssoidea	F	Fused to glycosyltransferase	KIF29242.1
Hassallia byssoidea	H	Fused to phosphoribosyl transferase	KIF29244.1
Theioarchaea archaeon	C	Discrete	KYK35486.1
Bathyarchaeota archaeon	A	Discrete	OGD46518.1
Micromonospora rifamycinica	D	Fused to carbamoyltransferase	WP_067301990.1
Bulkholderia sp. AU15512	A	Fused to iron redox enzyme	OXI24931.1
Nocardia sp. CS682	C	Fused to heme-oxygenase enzyme	QBS40287.1
Nocardia sp. CS682	D	Fused to iron redox enzyme	QBS40286.1
